# Supplementary material for: Make My Day: primary prevention of stroke using engaging everyday activities as a mediator of sustainable health – a randomised controlled trial and process evaluation protocol
Source: BMJ Open. 2023 Dec 6;13(12):e072037. doi: 10.1136/bmjopen-2023-072037 (PMC10711911; doi:10.1136/bmjopen-2023-072037)
Supplement: Supplementary data [file bmjopen-2023-072037supp001.pdf]

Summarized constent form: Make My Day- Primary prevention of stroke using engaging everyday activities as a mediator of sustainable health

INFORMATION TO PARTICIPANTS IN THE PROJECT

We are asking if you are interested in participating in a research project. In this document you will receive information about the project and what participation entails.

WHAT IS THE PROJECT ABOUT AND WHY DO YOU WANT ME TO PARTICIPATE?

You are asked to participate in the study after responded to the advertisement for the study. To participate in the project, you must have at least three modifiable risk factors for stroke (examples of risk factors are high blood pressure, stroke in the family, low level of physical activity, overweight, smoking, high alcohol intake and unhealthy eating habits) and be 55-75 years old.

You also need to be able to participate in a health promotion program in a primary healthcare centre in the region. The program consists of physical meetings in a group at a nearby healthcare center (6 meetings over 10 weeks) and registration of lifestyle habits in a mobile phone app.

The study aims to evaluate a stroke prevention program that addresses modifiable risk factors for stroke. The research principal for the project is KI meaning that KI is the organization responsible for the study. The study has been approved by the Swedish Ethical Review Authority.

WHAT WILL HAPPEN DURING THE PROJECT?

You will initially be contacted by a researcher. You will then receive additional information about the study and be able to ask questions about what it means to participate.

If you want to participate in the study, you will meet with a researcher on three occasions to answer questions (at the start of the study, after eleven weeks and twelve months after the start of the study). You will also be able to answer questions via surveys digitally at home at your own pace. The questions will be about different aspects of your daily life with a focus on health. Each event on site will take about 1 hour and can take place either via a physical meeting or online. You will be offered to wear an activity tracker to measure your activity level. The activity tracker will record your physical movements (e.g., how long you spend in a sitting or standing position).

After the first meeting, you will be randomized to be a control for the study or to be in the prevention group. If you are randomized to the prevention group, you will be offered to participate in the 10-week prevention program at a healthcare center. We cannot control the randomization. You will be contacted after the randomization has been completed to find out which group you belong to.

If you are part of the prevention program, you will be asked to answer questions and to tell us how participating in the prevention program has worked for you. You will also be asked to identify a close relative who could consider answering a survey and being interviewed. You will not be excluded from participation in the study if you do not have a relative, it is not a requirement to be able to participate.

POSSIBLE CONSEQUENCES AND RISKS OF PARTICIPATING IN THE PROJECT

Participating in the project should not pose any risk to you. If you should experience any kind of discomfort during or after the completion of the questionnaires or measurements, you are asked to

discontinue your participation. If you experience any discomfort or ill health during the study, you are asked to contact your healthcare center and then contact the responsible researcher. The healthcare center to which you belong will offer customary support to you if needed throughout the course of the study (i.e., the customary support via your healthcare center is not replaced by study participation).

WHAT HAPPENS TO MY DATA?

A researcher will collect and record information about you. Your answers and your results will be processed so that unauthorized persons cannot access them. No personal information that can be linked to you will be used, analyzed, or provided to a third party. The information is protected by regulations on confidentiality, which means that no unauthorized person may access the information. According to the EU's data protection regulation (GDPR), you have the right to access, free of charge, the information about you that is handled in the study and where we got it from, and if necessary to have any errors corrected. If you are dissatisfied with the way your personal data is processed, you have the right to lodge a complaint with the Swedish Authority for Privacy Protection.

HOW DO I GET INFORMATION ABOUT THE RESULT OF THE PROJECT?

During data collection sessions, you will be informed about your own test results. The study results will be presented at group level in reports, conferences and in scientific publications.

INSURANCE AND COMPENSATION

When participating in medical research you are covered by patient insurance, which is based on the Patient Injury Act (SFS 1996:799). No compensation is paid for participation in the study, for example no compensation is paid for lost income.

PARTICIPATION IS VOLUNTARY

Your participation is voluntary, and you can choose to cancel your participation at any time. If you choose not to participate or wish to cancel your participation, you do not need to state why, and it will not affect your future care or treatment.

RESPONSIBLE FOR THE PROJECT

Those responsible for the project are xxx:

Patomella A-H, *et al. BMJ Open* 2024; 13:e072037. doi: 10.1136/bmjopen-2023-072037

Summary of consent form: Make My Day

Consent to participate in the study

I have received oral and/or written information about the study and have had the opportunity to ask questions. I get to keep the written information.

• I agree to participate in the project: Stroke prevention – Development and evaluation of a person-centered, ICT-based intervention that supports a healthy activity pattern in everyday life in people who have an increased risk of suffering from a stroke

Location and Date	Signature
	Print name

Patomella A-H, *et al. BMJ Open* 2024; 13:e072037. doi: 10.1136/bmjopen-2023-072037
