# Supplementary material for: Make My Day: primary prevention of stroke using engaging everyday activities as a mediator of sustainable health – a randomised controlled trial and process evaluation protocol
Source: BMJ Open. 2023 Dec 6;13(12):e072037. doi: 10.1136/bmjopen-2023-072037 (PMC10711911; doi:10.1136/bmjopen-2023-072037)
Supplement: Author's manuscript [file bmjopen-2023-072037.draft_revisions.pdf]

BMJ Open is committed to open peer review. As part of this commitment we make the peer review history of every article we publish publicly available.

When an article is published we post the peer reviewers' comments and the authors' responses online. We also post the versions of the paper that were used during peer review. These are the versions that the peer review comments apply to.

The versions of the paper that follow are the versions that were submitted during the peer review process. They are not the versions of record or the final published versions. They should not be cited or distributed as the published version of this manuscript.

BMJ Open is an open access journal and the full, final, typeset and author-corrected version of record of the manuscript is available on our site with no access controls, subscription charges or pay-per-view fees (http://bmjopen.bmj.com).

If you have any questions on BMJ Open's open peer review process please email info.bmjopen@bmj.com

BMJ Open

Make My Day: Primary prevention of stroke using engaging everyday activities as a mediator of sustainable health—a randomised controlled trial and process evaluation protocol

Journal:	*BMJ Open*
Manuscript ID	bmjopen-2023-072037
Article Type:	Protocol
Date Submitted by the Author:	20-Jan-2023
Complete List of Authors:	Patomella, Ann-Helen; Karolinska Institutet, Department of Neurobiology, Care Sciences and Society; Academic Primary Health Care Centre<br>Guidetti, Susanne; Karolinska Institutet, Department of Neurobiology, Care Sciences and Society; Karolinska University Hospital<br>Hagströmer, Maria; Karolinska Institute, Department of Neurobiology Care Sciences and society; Academic Primary Health Care Centre<br>Olsson, Christina; Academic Primary Health Care Centre, ; Karolinska Institutet, Department of Neurobiology, Care Sciences and Society<br>Jakobsson, E; Karolinska Institutet, Department of Neurobiology, Care Sciences and Society<br>Nilsson, Gunnar; Academic Primary Health Care Centre; Karolinska Institutet, Department of Neurobiology, Care Sciences and Society<br>Åkesson, Elisabet; Karolinska Institutet, Department of Neurobiology, Care Sciences and Society; Stockholms Sjukhem FoUU<br>Asaba, Eric; Karolinska Institutet, Department of Neurobiology, Care Sciences and Society; Stockholms Sjukhem FoUU
Keywords:	Stroke < NEUROLOGY, PREVENTIVE MEDICINE, REHABILITATION MEDICINE

SCHOLARONE™
Manuscripts

Make My Day: Primary prevention of stroke using engaging everyday activities as a mediator of sustainable health—a randomised controlled trial and process evaluation protocol

Patomella Ann-Helen, ann-helen.patomella@ki.se, (ORCID 0000-0003-2667-4073)[1, 2]

Guidetti Susanne, Susanne.guidetti@ki.se, (ORCID 0000-0001-6878-6394) [1,3]

Hagströmer Maria, maria.hagstromer@ki.se, (ORCID 0000-0002-4607-8677) [1, 2]

Olsson Christina B, christina.b.olsson@regionstockholm.se, (0000-0003-1300-7765) [2,4]

Jakobsson Elin, elin.jakobsson@ki.se, (ORCID 0000-0001-9756-1354) [1]

Nilsson Gunnar, gunnar.nilsson@ki.se, (ORCID 0000-0001-7811-7602) [2,5]

Åkesson Elisabet, elisabet.akesson@ki.se, (ORCID 0000-0002-8227-9118) [6,7]

Asaba Eric, eric.asaba@ki.se, (ORCID 0000-0002-6910-3468)[1,7]

Affiliation:

[1]Department of Neurobiology, Care Sciences and Society, Division of Occupational Therapy, Karolinska Institutet, Stockholm, Sweden

[2] Academic Primary Health Care Centre, Region Stockholm, Stockholm, Sweden

[3] Women´s Health and Allied Health Professionals Theme, Medical Unit Occupational therapy and Physiotherapy, Karolinska University Hospital

[4] Department of Neurobiology, Care Sciences and Society, Division of Physioterapy Therapy, Karolinska Institutet, Stockholm, Sweden

[5] Department of Neurobiology, Care Sciences and Society, Division of Family Medicine and Primary Care, Karolinska Institutet, Stockholm, Sweden

[6] Department of Neurobiology, Care Sciences and Society, Division of Neurogeriatrics, Karolinska Institutet, Stockholm, Sweden

[7] R&D Unit, Stockholms Sjukhem, Stockholm, Sweden

For peer review only - http://bmjopen.bmj.com/site/about/guidelines.xhtml

Corresponding author:
Ann-Helen Patomella, Department of Neurobiology, Care Sciences and Society, Division of Occupational Therapy, Karolinska Institutet, Stockholm, Sweden
Email address: ann-helen.patomella@ki.se
Phone number: +46 852483726

* I, the Submitting Author has the right to grant and does grant on behalf of all authors of the Work (as defined in the below author licence), an exclusive licence and/or a non-exclusive licence for contributions from authors who are: i) UK Crown employees; ii) where BMJ has agreed a CC-BY licence shall apply, and/or iii) in accordance with the terms applicable for US Federal Government officers or employees acting as part of their official duties; on a worldwide, perpetual, irrevocable, royalty-free basis to BMJ Publishing Group Ltd ("BMJ") its licensees and where the relevant Journal is co-owned by BMJ to the co-owners of the Journal, to publish the Work in BMJ Open and any other BMJ products and to exploit all rights, as set out in our licence.

The Submitting Author accepts and understands that any supply made under these terms is made by BMJ to the Submitting Author unless you are acting as an employee on behalf of your employer or a postgraduate student of an affiliated institution which is paying any applicable article publishing charge ("APC") for Open Access articles. Where the Submitting Author wishes to make the Work available on an Open Access basis (and intends to pay the relevant APC), the terms of reuse of such Open Access shall be governed by a Creative Commons licence – details of these licences and which Creative Commons licence will apply to this Work are set out in our licence referred to above.

Word count: 3999
Protocol version: 18th of January 2023. Version 1.0
Trial Registration number: ClinicalTrials.gov Identifier: NCT05279508

For peer review only - http://bmjopen.bmj.com/site/about/guidelines.xhtml

Abstract

Introduction

The individual, societal and economic benefits of stroke prevention are high. Even though most risk factors can be reduced by changes to lifestyle habits, maintaining new and healthy activity patterns has been shown to be challenging.

The aim of the study is to evaluate the impact of an interdisciplinary team-based, mHealth-supported prevention intervention in primary health care (PHC). The intervention is mediated by engaging everyday activities that promote health. An additional aim is to describe a process evaluation that serves to increase knowledge about how the program leads to potential change by studying the implementation process and mechanisms of impact.

Methods and analysis

The study will be a randomised controlled trial including 104 persons at risk for stroke. Persons at risk of stroke (n =52) will be randomised to an mHealth-supported stroke prevention program. Controls will have ordinary PHC services. The 10-week programme will be conducted at PHC clinics, combining group meetings and online resources to support self-management of lifestyle change using engaging everyday activities as a mediator. Primary outcomes are stroke risk, lifestyle habits, and participation in health-promoting activities. Assessments will be performed at baseline and at follow-up (11 weeks and 12 months). Effects of the programme will be analysed using inferential statistics. Implementation will be analysed using qualitative and quantitative methods.

Ethics and dissemination

The study has been approved by the Swedish Ethical Review Authority. Study results will be disseminated in peer-reviewed journals and at regional and international conferences targeting mixed audiences.

For peer review only

Article Summary

Strengths of this study

- The robust randomised controlled trial (RCT) design, investigating the effects of the intervention programme for persons at risk of stroke.
- A process evaluation will provide rich data, useful for analysing the outcomes and the research process and for implementation in primary health care.
- The utilisation of engaging everyday activities as a mediator and goal for sustainable lifestyle change.

Limitations of this study

- A limitation is that primary outcomes are based on self-reported data. Therefore, a physical activity monitoring device will be used to track movement and activity.
- One of the inclusion criteria is motivation, which can skew the results and lower external validity.

For peer review only

For peer review only - http://bmjopen.bmj.com/site/about/guidelines.xhtml

Abstract

Introduction

The individual, societal and economic benefits of stroke prevention are high. Even though most risk factors can be reduced by changes to lifestyle habits, maintaining new and healthy activity patterns has been shown to be challenging.

The aim of the study is to evaluate the impact of an interdisciplinary team-based, mHealth-supported prevention intervention in primary health care (PHC). The intervention is mediated by engaging everyday activities that promote health. An additional aim is to describe a process evaluation that serves to increase knowledge about how the program leads to potential change by studying the implementation process and mechanisms of impact.

Methods and analysis

The study will be a randomised controlled trial including 104 persons at risk for stroke. Persons at risk of stroke (n =52) will be randomised to an mHealth-supported stroke prevention program. Controls will have ordinary PHC services. The 10-week programme will be conducted at PHC clinics, combining group meetings and online resources to support self-management of lifestyle change using engaging everyday activities as a mediator. Primary outcomes are stroke risk, lifestyle habits, and participation in health-promoting activities. Assessments will be performed at baseline and at follow-up (11 weeks and 12 months). Effects of the programme will be analysed using inferential statistics. Implementation will be analysed using qualitative and quantitative methods.

Ethics and dissemination

The study has been approved by the Swedish Ethical Review Authority. Study results will be disseminated in peer-reviewed journals and at regional and international conferences targeting mixed audiences.

For peer review only - http://bmjopen.bmj.com/site/about/guidelines.xhtml

Article Summary

Strengths of this study

- The robust randomised controlled trial (RCT) design, investigating the effects of the intervention programme for persons at risk of stroke.
- A process evaluation will provide rich data, useful for analysing the outcomes and the research process and for implementation in primary health care.
- The utilisation of engaging everyday activities as a mediator and goal for sustainable lifestyle change.

Limitations of this study

- A limitation is that primary outcomes are based on self-reported data. Therefore, a physical activity monitoring device will be used to track movement and activity.
- One of the inclusion criteria is motivation, which can skew the results and lower external validity.

For peer review only

INTRODUCTION

In Sweden, a health care reform programme entitled "Good Quality Local Health Care—Primary Care Reform" (1) is currently paving the way for a large-scale transformation. The reform targets primary health care (PHC) specifically, and is designed to proactively address and prevent illness at an early stage to reduce the burden of non-communicable diseases (NCD), like stroke. The reform is in line with the United Nations Sustainable Development Goals, urging governments to reduce premature mortality from NCDs by 1/3 by year 2030 through prevention and treatment (2). By the age of 55, the risk for stroke increases considerably and doubles each decade afterward (3,4). Although the incidence of stroke has decreased in the general population, trends show an increase in stroke incidence in young adults (5). In Sweden, stroke incidence has declined by over 40% the last 15 years (6). However, both globally and in Sweden, cerebrovascular diseases like stroke continue to be the most common cause of death and impairment (7).

The individual, societal, and economic benefits of stroke prevention are high. Many of the stroke risk factors are largely addressable; e.g., smoking, obesity, type 2 diabetes, hypertension, physical inactivity, and dietary intake. The benefits of a healthy lifestyle are clear (4,8), however, the long-term effect of lifestyle interventions are not (9,10). For example, the effectiveness of PHC-based physical activity interventions is inconclusive (11). There is evidence for short-term improvements, but there is a lack of evidence for long-term effects (9). A multi-factorial approach to stroke prevention is warranted. A systematic review showed that multifactorial lifestyle habit interventions have greater potential effect on reducing risk factors than single-factor interventions (12). Preliminary evidence exists from a Swedish trial on a multifactorial lifestyle counselling program (13) that improved physical activity and dietary habits and reduced smoking and stress; however, the study was not conducted in PHC and did not have a control group. mHealth (a term for the combination of eHealth services and smartphone technology (14)) presents possibilities for accessibility and affordability when developing health services (15, 16) making it an excellent tool for designing stroke prevention models (17).

Theoretical concept of the intervention program

For peer review only - http://bmjopen.bmj.com/site/about/guidelines.xhtml

The program is a complex intervention, and the Medical Research Council (MRC) guidance for developing and evaluating complex interventions (18) has been utilised in the design of the study. MRC suggest several key elements and stages; Development, Feasibility/piloting, Evaluation, and Implementation. This randomised controlled trial (RCT) evaluates the effectiveness and implementation of the Make My Day (MMD) programme, and is based on already-completed or ongoing studies concerning development and feasibility.

Engaging everyday activity

In the MMD programme, engaging everyday activities (EEA) are seen as the means and goal for changing and sustaining a healthy lifestyle in the intervention program, and while it is not a new concept, it has not been studied in relation to changing lifestyle habits to prevent NCDs. EEA is defined as a special type of activity that: is based on individual experience, filled with meaning, and gives a sense of intense participation and enthusiasm to the individual (19, 20). Examples from previous studies show that EEA can include a variety of activities such as working, playing computer games, and reading books. We recently showed that an intervention programme targeting cardiovascular disease prevention could benefit from incorporating health promoting EEA (21). The complexity of changing lifestyle habits has been described as a paradox between EEA and health (22). Even though EEA is subjectively meaningful and engaging to an individual, it might have an arguably negative impact on health; for example, engagement in sedentary activity or in unhealthy behaviours (22). In the current project, EEAs are seen as having the potential to change everyday activity patterns, and when carefully designed (for example, listening to an audiobook while taking a walk), incorporate a routine that promotes sustainable health among persons at risk for stroke. Studies have shown that promoting EEAs can have positive health impacts for older adults (23–25). Studies on populations that live lives that incorporate EEAs combined with moderate-intensity physical activities and a healthy diet indicate a strong relation to wellbeing, longevity, and cultural context (4,5).

Current state-of-the art in stroke prevention suggest the need for a multifactorial PHC intervention that address modifiable risk factors for stroke based on individual needs

For peer review only - http://bmjopen.bmj.com/site/about/guidelines.xhtml

and engagement in everyday activities that promote health with the support of a mHealth service.

Objectives of the proposed study

The main aim of the study is to evaluate the impact of an inter-disciplinary team-based, mHealth-supported prevention intervention in primary health care (PHC) — mediated with engaging everyday activities that promote health—to decrease stroke risk (primary outcome), and increase participation in engaging everyday activities and Health Related Quality of Life (HRQoL). The aim is also to describe a process evaluation that serves to increase knowledge about how the program leads to potential change by studying the implementation process and mechanisms of impact.

Hypothesis

We hypothesise that the Make My Day (MMD) intervention programme is more beneficial than regular PHC services (control group) in decreasing stroke risk (primary outcome). We also hypothesised that MMD is more beneficial than regular PHC in increasing a) participation in health promoting EEAs, and b) HRQoL.

METHODS AND ANALYSIS

Design

The study will be a randomised, assessor-blinded, controlled trial of persons at risk for stroke. The process evaluation answers questions as to what interventions were delivered and how by combining qualitative interviews and descriptive quantitative data on the implementation, casual mechanisms, and contextual variation (26).

Study setting

The study will be conducted in close collaboration with four PHC clinics in the Stockholm area (different parts of Stockholm to represent a diversity in geographical area). PHC clinics in this study are rehabilitation units involving dietitians, physiotherapists, and occupational therapists. In Region Stockholm, rehabilitation units are often both organisationally and geographically separate from general practitioner (GP) primary health care clinics. These primary health care rehabilitation units have an agreement with the County Council in Stockholm, and are available for patients to choose from without the need of a referral for treatment by certified

For peer review only - http://bmjopen.bmj.com/site/about/guidelines.xhtml

For peer review only

physiotherapists, occupational therapists, and dieticians. PHC services are publicly funded in Sweden.

Sample size and power considerations

The primary outcome is stroke risk. Based on data from a case study (27), we calculate a decrease of at least one level of stroke risk (e.g., moving from high risk to medium risk in the Stroke Risk Score Card (28)) with a standard deviation of 1.5, statistical power of 80%, with two-railed α =0.05. Under these assumptions, the required sample size was 35 in each group. The Stroke Risk Score Card was developed as an easy-to-use self-assessment tool by the National Stroke Association in the United Kingdom (28). The tool has been used previously in a few studies to detect risk factors of stroke (27,28) and in a recently-finished pilot study conducted in the research group (publication in manuscript). Since the Stroke Risk Score Card has not been sufficiently tested psychometrically, power calculations were added for participation in EEAs, and a newly developed assessment tool for stroke risk was added as a primary outcome measure (not possible to use in power calculations, since no previous data were available). Power to detect a clinically important difference on participation (performance and satisfaction) in EEAs of two points (as measured with the Canadian Occupational Performance Measure, COPM (29)), requires 40 participants in each group. To safeguard against dropouts (a maximum 30% dropout rate is assumed), 104 participants will be enrolled. In total, five groups (at 3-4 different PHC clinics), each consisting of approximately 10–12 participants will receive the prevention program.

Participants: Recruitment and eligibility criteria

Persons at risk of stroke will be included in the project and participants will be recruited via advertisements in social media, a webpage, and flyers at PHCs. A stroke risk online screening survey will be used to find eligible participants. Inclusion criteria for the study are a) three or more risk factors deemed ‘high risk’ using the Stroke Risk Score Card, b) motivation for lifestyle change and to participate in a digital lifestyle intervention (including use of a smart phone), d) aged between 55–75 years old, and without a diagnosis of dementia or cognitive impairment hindering participation. Exclusion criteria include having previously had a stroke or a trans-ischemic attack diagnosis, lack of understanding of the Swedish language, and not

For peer review only - http://bmjopen.bmj.com/site/about/guidelines.xhtml

being able to use a mobile phone application. A total sample of n =104 participants (persons at risk of stroke), divided into two arms (52+52) for intervention and controls is estimated. Block randomisation will be utilised with a block size of four (25). Allocation will be done following baseline assessment by a researcher not involved in data collection nor intervention. The assessors of outcomes will be blinded to allocation until the end of the study.

In addition, next of kin to persons at risk for stroke are also invited to answer questions (survey and group interview) regarding support of their relative and of their own health. Persons at risk for stroke who do not have a next of kin to answer the survey will not be excluded. PHC professionals that have been trained and delivered the interventions programme will be additional participants in the process evaluation of the study. Stakeholders (such as leaders at the involved PHC clinics) will be invited to individual interviews.

Informed consent

Written informed consent will be obtained from all participants (persons at risk, their next of kin, and PHC staff and stakeholders) at the start of recruitment. Information about the study will be given in written and verbal forms during meetings with research staff. Persons at risk will be asked to identify a next of kin that will be asked to participate by the researchers.

Make My Day—a stroke-prevention program

The MMD intervention program enables healthy activity patterns and aims to reduce the risk of stroke by means of four strategies: a) the incorporation of health-promoting EEAs, b) the use of mobile phone technology (mHealth) to increase health literacy, and awareness of current habits and fostering self-management, c) setting realistic goals that form new habits that prompt conscious decisions for healthy choices and habits, and d) sharing experience in a learning environment.

Duration and specific content of the intervention programme

The MMD stroke intervention programme is a 10-week group programme consisting of five sessions over the first five weeks, followed by a sixth booster session five weeks later. During the intervention, participants will work actively on self-chosen

For peer review only - http://bmjopen.bmj.com/site/about/guidelines.xhtml

goals, EEAs, and habits to change behaviour and lifestyle. A mobile phone app will be used by participants throughout the 10 weeks, supporting their awareness of current lifestyle habits and everyday activities. To form new habits, common situations will be used to cue behaviour change, like seeing an elevator and looking for the staircase, prompting health-promoting behaviour, and making a conscious decision to walk the stairs (30). The continuation of a change process is expected from the participants following the 10-week program period, and strategies for self-management are anticipated.

Each session (90 min) has a theme and includes some type of activity such as exercise, making a light snack, or taking a walk. Group dynamics and personal experience are used to reflect on EEAs in relation to health, doing and future goals. The sessions and content, presented in Table 1, are delivered by a trained health professional; for example an occupational therapist, physiotherapist, or dietician.

The health care professionals who will provide the MMD program/intervention will participate in structured education specially designed for this program. This education will be given in an on-site and digital combination on three occasions, and will be held by two research team members with extensive experience in pedagogy and in the research protocol. In addition, the health professionals will have access to a digital educational platform with rich and varied material, and all material to be used during the 10-week programme. To avoid contamination, the health professionals are instructed to not deliver the 10-week program to other patients during the research period.

Table 1: Summary of session themes, concepts, and activities supporting a change process

Week	Session theme	Profession	Concepts	Activity
1	**1:** Risk factors for stroke and engaging everyday activities	Occupational therapist	Health literacy concerning stroke risk, engaging activities, change process, expectations	Peer interview on engaging activities. Learn how to register in the app
2	**2:** Physical activity	Physiotherapist	Physical activity, physical inactivity	Try a physical group exercise class at a gym
3	**3:** Diet and health	Dietician	Dietary routines and change	Food lab—prepare and test; e.g., healthy snacks/sandwiches

For peer review only - http://bmjopen.bmj.com/site/about/guidelines.xhtml

4	**4:** Balanced everyday life	Occupational therapist	Activity balance and stressors	Relaxation—such as medical yoga or meditation
5	**5:** The meaning of healthy habits, routines, and activity patterns	One of the team members	Current and desired routines/habits, activity patterns and resources	Walk-and-talk—e.g., in a forest or a historical walk in the city
10	**6:** Booster session: Evaluation and the road ahead	Occupational therapist	Self-management, sustainability, view of the self, social support, revisiting goals/new goals, and social aspects of health	Preparing healthy snacks and group reflection on the program

The mobile phone app

The app for the pilot project was produced collaboration with ScientificMed Tech AB (now part of Cuviva AB) (http://www.scientificmed.com), and for the current project the app has been modified based on previous experiences of the users and the researchers. A workshop with the pilot study participants, researchers and the company showed that the participants wanted the app to be more tailored to their needs with a more user-friendly interface. Both the researchers and the users wanted feedback to be relevant and tailored to the users progress thus supporting change and awareness. As with the previous version, the new version of the app includes six domains for registering daily activities, experiences, and behaviours (see Figure 1 for examples from the app): my goals (goal achievements on three pre-set goals); physical activity and steps (step counts, 24-hour time use in relation to exercise, moderately intense activities, sleep, sedentary activities, and other activities); engaging activities (participating in health-promoting EEAs); tobacco and alcohol use (consumption); stress (perceived time-pressure); and dietary habits (consumption of fruits/vegetables, breakfast, fish and snacks—not included in the figure). Domains are based on modifiable risk factors for stroke, as presented by the American Heart Association (3), with the addition of health-promoting EEAs and stress reduction.

-Insert Figure 1 here

Data collection

Persons at risk for stoke

Data collection with a research assistant starts with an individual meeting (baseline) with all eligible participants, just before the meeting (T minus 2 days), during which participants are sent a link to an online survey for collecting self-reported measures.

For peer review only - http://bmjopen.bmj.com/site/about/guidelines.xhtml

During baseline assessment, all participants (including controls) will be informed of their stroke risk factors. Motivational interviewing techniques will be used to identify three problem areas in relation to lifestyle habits and stroke risk factors, and these areas will be used to formulate three lifestyle change goals. Allocation (randomisation) will be done following baseline assessment. Allocation sequences will be done by an independent researcher not involved in data collection or intervention. The researchers who are assessors of outcomes will be blinded to allocation until the end of the study. The assessments measuring primary and secondary outcomes will be collected at baseline, at follow-up (11 weeks), and at 12 months; see Table 2. Demographic data will be collected at baseline. Process data will be collected continuously. Controls will be offered standard care by PHCs as needed during the 12 months study period.

Outcome data

Outcome assessment methods were carefully chosen to assure methods that are valid and reliable and will capture change. The primary outcome measure is risk for stroke, measured by the Swedish version of the *Stroke Riskometer* (30,31) and the Stroke Risk Score Card (27, 28). Secondary outcomes include participation in health-promoting everyday activities, measured by the *Canadian Occupational Performance Measure*, (COPM) (32), and self-rated health measured using *LiSat-11* (33) and *EQ-5D* (34). Other measures are lifestyle habits (measured using the updated *Swedish Lifestyle Survey*, Levnadsvaneenkäten) (35), and activity patterns, as measured using the Swedish version of the *Productivity Pleasure and Restoration Profile* (36, 37). Survey data will be gathered for health literacy of stroke risk (38), experiences of time pressure (stress), cost effectiveness (e.g., self-reported sick leave; health care utilisation, and use of medication), readiness and motivation for change (39), current mobile phone use, and mapping out engaging everyday activities. Habitual physical activity will be measured using the activPAL® micro activity monitor (PAL Technologies Ltd., Glasgow, UK) (40). The activPAL is a small device which provides information on position and acceleration of the body. The monitor is attached to the thigh and will be worn for five consecutive days after baseline and at follow-up (11 weeks and 12 months). Outcomes from the monitor are 1) time spent sitting/lying, standing, stepping, 2) numbers of step counts, and 3) sit-to-stand transitions.

For peer review only - http://bmjopen.bmj.com/site/about/guidelines.xhtml

For peer review only

Outcome data- next of kin

Data from next of kins to participants in the intervention group will be collected via an online survey after the intervention period. The survey will include demographic measures of health and questions on their view of the program and the support they have given their kin.

Process data

The process evaluation will illuminate casual mechanisms and help identify factors that are associated with variation in outcomes, such as contextual and external factors (26). Process data include both qualitative and quantitative descriptive data, including logbooks from PHC staff (notes taken during delivery of the program), course evaluations from the web-based staff training, and semi-structured exit interviews with participants at risk for stroke and their next of kin, see Table 2. Fidelity will be evaluated as the extent to which the programme was delivered as expected. Dose will be assessed as the quantity of the implemented intervention. Adaptation, such as changes made to fit different PHC settings, will be collected during interviews with PHC staff. Reach will be assessed regarding how many eligible patients signed up and how many completed the MMD program. In addition, adverse events will be registered. Context includes external factors that may act as a barrier or facilitator to the implementation itself and to the interventions' effects. Assessing barriers and facilitators to program implementation will also involve evaluating program feasibility; i.e., the extent to which stakeholders regard the MMD as satisfactory in terms of content and complexity/difficulty.

Data will be managed using an online software called RedCap (https://www.project-redcap.org/) in combination with a local data management system.

Participant timeline

Participant enrolment will start in April 2022, and the last groups' 12-month follow-up will occur in March 2024 (marking the end of the study).

Table 2: Summary of measures to be collected

	**Instrument and scale**	**Time points**

For peer review only - http://bmjopen.bmj.com/site/about/guidelines.xhtml

**Primary outcome measures from persons at risk for stroke:**		
Stroke risk	The Stroke Riskometer*, the Stroke risk score card*	t1, t2, t3
**Secondary outcome measures:**		
Participation in everyday activities	COPM, PPR profile*	t1, t2, t3
Physical activity (habitual)	ActivPal	t1, t2, t3
Life satisfaction	EQ-5D*, LiSat-11*	t1, t2, t3
Lifestyle habits	The Swedish Lifestyle Habits Questionnaire*	t1, t2, t3
**Demographics and measures:**		
Age	Year*	t1
Gender	Male/Female/Other*	t1
Ethnic background	Mother tongue*, place of birth*	t1
Height	Cm	t1
Weight	Kg*	t1, t2, t3
Living situation	Living alone or not*	t1, t3
Yearly income	In Swedish crona*	t1, t3
Employment status	Part-time, full-time, sick-leave, unemployed, student, retired*	t1, t3
Level of education	Years of education*	t1
Blood pressure	mmHg	t1, t2, t3
Health literacy	Knowledge of stroke	t1, t2, t3
Motivation for change	Self-reported, ordinal scale	t1
Cost effectiveness	Self-reported sick-leave and absence from work past six months; health care utilisation past six months; Use of medication	t1, t2, t3
**Experiences of next of kin:**	Self-reported health and support*	t2, t3
**Process data:**		
Fidelity and adaptations	Interviews with interventionists on delivery of intervention. Log-books from interventionists.	t2
Dose	Log-books from interventionists.	t2

For peer review only - http://bmjopen.bmj.com/site/about/guidelines.xhtml

t1 =Baseline; t2 =One week following intervention ending; t3 =12 months follow-up post baseline
* = measures collected via an online survey

Data Analysis Plan

Outcomes on effects

The characteristics of all persons at risk for stroke at inclusion, and outcomes at 11 weeks and 12 months after inclusion, will be presented with descriptive statistics. The treatment effects in the RCT study will be analysed on an intention-to-treat basis, with randomised participants retaining their original allocated group, and measured as differences between groups at follow-up and at 12 months considering plausible confounders. Outcome data will be examined for outliers, normality, and missing data. Analyses of covariance will be used for continuous outcomes, with baseline values as covariates. Logistic regression analyses will be used for dichotomous outcomes. The level of significance will be set at $p \leq 0.05$ and the confidence level at 95%. We will use SPSS to analyse the data. Analyses will provide results for the relative effectiveness of the intervention program. The results will be reported accordance to CONSORT recommendations for reporting randomised controlled trials on non-pharmacological treatments (41) and the protocol has been reported according to the SPIRIT reporting guidelines (42).

Process evaluation

A mixed method approach where qualitative and quantitative data is integrated will be used to answer how the implementation process and potential mechanisms of impact can explain the outcomes of the MMD intervention.

Data collected from surveys, logbooks on recruitment and dropout, and logs from the app registrations will be entered, analysed, and summarised. Descriptive statistical analyses will be conducted to report on the study’s feasibility: recruitment, drop-outs, retention rate, and adherence. Data from app registrations will be used to report on feasibility and usability. Qualitative interviews will be transcribed verbatim and analysed using thematic qualitative analyses.

For peer review only - http://bmjopen.bmj.com/site/about/guidelines.xhtml

Patient and Public Involvement

Persons at risk for stroke participating in previous studies have been active in the development of material and research processes.

Discussion

Several NCDs share the same risk factors as stroke, and an intervention program has the potential to address other NCDs and health in general, and should overlap with other health-promoting strategies (3). In the proposed study we will evaluate the MMD program in regard to decreasing stroke risk by using broad strategies and addressing multiple factors of relevance. The theoretical base of the protocol is grounded on EEAs as the mediator and goal for decreasing stroke risk and sustaining personally-relevant healthy living habits. It is important to note that the concept of personal relevance can mean that in a total week of different activities, some engaging activities can potentially be considered unhealthy (i.e., unhealthy eating activity), but the overall pattern of participation in EEA could be designed to include health-promoting EEA as this study promotes. The paradoxicality of EEAs is that the feeling of being engaged can be just as important for health and wellbeing as being physically active (43). Living habits thus needs to be seen as part of a broader life context, in which health and EEAs are continuously renegotiated and thus need to be regularly reassessed within the context of each person’s life situation.

During the COVID-19 pandemic there was a strong increase in online primary health care consultations in Sweden, especially for younger patients with high economical and educational backgrounds who were born in Sweden; meanwhile the older population sought less care and preferred face-to-face consultations (44). Although there is a possibility to deliver the MMD intervention programme completely online (no physical meetings), we have decided to run the programme meetings face-to-face. During the 12-month follow-up of the pilot study, which occurred at the end of 2020, participants rated physical meet-ups (the possibility of exchanging experiences with other at-risk persons and group leaders) as highly valued; which is in line with previous studies that showed that a blended intervention approach can be efficient compared to only online or on-site intervention (45). However, whether multiple face-

For peer review only - http://bmjopen.bmj.com/site/about/guidelines.xhtml

to-face consultations (doses) would be the most efficient is not clear, and is one of the questions for the process evaluation.

The possible limitation of the study will be the reliability of self-reported measures, and there is a risk of bias since reporting might not be accurate, therefore measures such as activPAL, BMI, and blood pressure are complementing the assessments. Although we have planned for five days of activPAL wear (the recommendation is seven days), participants will wear these 24/7, and we will monitor data loss. External validity of the outcomes could be flawed, due to a recruitment process mainly benefiting highly-motivated persons at risk of stroke, and the risk of dropouts in less-motivated participants. Ethical dilemmas include that controls are not being supported in the same way as the intervention group and that the recruitment methods could be skewed and fail to reach out to vulnerable groups in society (lower SES) at risk for stroke. The strength of the study lies in the robustness of the RCT design, the process evaluation, and the interprofessional collaboration in a clinical PHC context. The data from the process evaluation will increase and ease the possibility of implementation of a prevention program for NCDs in PHC. The risk of contamination between control and intervention is deemed minimal, as participants are recruited via social media in a large city.

ETHICS AND DISSEMINATION

An approval from the Swedish Ethical Review Authority, Sweden has been granted (Ref. numbers. 2015/834-31, 2016/2203-32, 2019/01444 and 2021-05902-02). Data management will be complying with the general data protection regulation, GDPR, and all data will be stored securely to protect the confidentiality. Participation in the study is not expected to lead to health risks or complications, and potential health consequences will be monitored. Participant who experiences any health-related problems during the study will be guided to contact their GP. Participants may choose to interrupt their participation in the study at any time. Researchers can also discontinue a participant’s participation based on health issues, or reasons that might jeopardise that person’s safety. Reasons for interruption will be recorded.

For peer review only - http://bmjopen.bmj.com/site/about/guidelines.xhtml

The findings of the study will be published in peer-reviewed journals, and the results will be disseminated to participants, the public, PHC staff, and decision-makers through national and international conferences, as well as study-specific web pages.

AUTHOR CONTRIBUTIONS

AHP, EA, and SG conceived the original idea and outline of the study. AHP is implementing the protocol in primary healthcare settings, with regular dialog and review by CO, EÅ, GN, EA, and SG. CO, MH, GN, and EÅ contributed to the design of the study. AHP wrote the study protocol, together with EA and EJ. All authors discussed and commented on draft versions and approved the final version.

FUNDING STATEMENT

This work was funded by grants from FORTE, grant number 2020-00175. This funding source had no role in the design of this study and will not have any role during its execution, analyses, interpretation of the data, or decision to submit results.

COMPETING INTERESTS

The authors declare that they have no competing interests.

For peer review only

For peer review only - http://bmjopen.bmj.com/site/about/guidelines.xhtml

REFERENCES

1. Government S. Good quality, local health care—A primary care reform). http://www.sou.gov.se/wp-content/uploads/2018/06/Summary-SOU-2018.39_Final2.pdf; 2018. Report No.: SOU 2018:39.

2. Assembly. UNG. Transforming our world: The 2030 Agenda for sustainable development. 2016 2022-11-30. Available from: https://documents-dds-ny.un.org/doc/UNDOC/GEN/N15/291/89/PDF/N1529189.pdf?OpenElement

3. Habner J. Preventing stroke: Uneven progress. A global policy research programme. 2017.
4. Goldstein LB, Bushnell CD, Adams RJ, Appel LJ, Braun LT, Chaturvedi S, et al. Guidelines for the primary prevention of stroke: a guideline for healthcare professionals from the American Heart Association/American Stroke Association. Stroke. 2011;42(2):517–84.
5. Béjot Y, Bailly H, Durier J, Giroud M. Epidemiology of stroke in Europe and trends for the 21st century. La Presse Médicale. 2016;45(12, Part 2):e391–e8.
6. Welfare TNBoHa. Statistics on Stroke 2020. 2021. Contract No.: Art no. 2021-12-7644.
7. Organization) WWH. The top 10 causes of death 2020 [Available from: https://www.who.int/news-room/fact-sheets/detail/the-top-10-causes-of-death.
8. Chiuve SE, Rexrode KM, Spiegelman D, Logroscino G, Manson JE, Rimm EB. Primary prevention of stroke by healthy lifestyle. Circulation. 2008;118(9):947–54.
9. Eakin EG, Glasgow RE, Riley KM. Review of primary care-based physical activity intervention studies: effectiveness and implications for practice and future research. Database of Abstracts of Reviews of Effects (DARE): Quality-assessed Reviews [Internet]: Centre for Reviews and Dissemination (UK); 2000.
10. Weiss EC, Galuska DA, Khan LK, Gillespie C, Serdula MK. Weight regain in US adults who experienced substantial weight loss, 1999–2002. American journal of preventive medicine. 2007;33(1):34–40.
11. Hillsdon M, Thorogood M, White I, Foster C. Advising people to take more exercise is ineffective: a randomized controlled trial of physical activity promotion in primary care. Int J Epidemiol. 2002;31(4):808–15.
12. Sisti L, Dajko M, Campanella P, Shkurti E, Ricciardi W, De Waure C. The effect of multifactorial lifestyle interventions on cardiovascular risk factors: a systematic review and meta-analysis of trials conducted in the general population and high risk groups. Preventive medicine. 2018;109:82–97.
13. Lönnberg L, Ekblom-Bak E, Damberg M. Improved unhealthy lifestyle habits in patients with high cardiovascular risk: results from a structured lifestyle programme in primary care. Upsala journal of medical sciences. 2019;124(2):94–104.
14. Adibi S. Mobile Health: A Technology Road Map. Adibi S, editor: Springer; 2015.
15. Zonneveld M, Patomella A-H, Asaba E, Guidetti S. The use of information and communication technology in healthcare to improve participation in everyday life: a scoping review. Disability and Rehabilitation. 2019:1–8.
16. Chow CK, Ariyarathna N, Islam SM, Thiagalingam A, Redfern J. mHealth in Cardiovascular Health Care. Heart Lung Circ. 2016;25(8):802–7.
17. Owolabi MO, Thrift AG, Mahal A, Ishida M, Martins S, Johnson WD, et al. Primary stroke prevention worldwide: translating evidence into action. The Lancet Public Health. 2021.
18. Skivington K, Matthews L, Simpson SA, Craig P, Baird J, Blazeby JM, et al. Framework for the development and evaluation of complex interventions: gap analysis, workshop and consultation-informed update. 2021.

For peer review only - http://bmjopen.bmj.com/site/about/guidelines.xhtml

19. Jonsson H. A new direction in the conceptualization and categorization of occupation. Journal of occupational science. 2008;15(1):3–8.
20. Jonsson H, Josephsson S, Kielhofner G. Narratives and experience in an occupational transition: a longitudinal study of the retirement process. Am J Occup Ther. 2001;55(4):424–32.
21. Mälstam E, Asaba E, Åkesson E, Guidetti S, Patomella A-H. 'Weaving lifestyle habits': Complex pathways to health for persons at risk for stroke. Scandinavian journal of occupational therapy. 2021:1–13.
22. Asaba E, Bergström A, Patomella AH, Guidetti S. Engaging occupations among persons at risk for stroke: A health paradox. Scandinavian journal of occupational therapy. 2020:1–10.
23. Clark F, Azen SP, Zemke R, Jackson J, Carlson M, Mandel D, et al. Occupational therapy for independent-living older adults. A randomized controlled trial. JAMA. 1997;278(16):1321–6.
24. Clark F, Jackson J, Carlson M, Chou CP, Cherry BJ, Jordan-Marsh M, et al. Effectiveness of a lifestyle intervention in promoting the well-being of independently living older people: results of the Well Elderly 2 Randomised Controlled Trial. J Epidemiol Community Health. 2012;66(9):782–90.
25. Mountain G, Windle G, Hind D, Walters S, Keertharuth A, Chatters R, et al. A preventative lifestyle intervention for older adults (lifestyle matters): a randomised controlled trial. Age Ageing. 2017;46(4):627–34.
26. Moore GF, Audrey S, Barker M, Bond L, Bonell C, Hardeman W, et al. Process evaluation of complex interventions: Medical Research Council guidance. BMJ. 2015;350.
27. Patomella A-H, Farias L, Eriksson C, Guidetti S, Asaba E. Engagement in Everyday Activities for Prevention of Stroke: Feasibility of an mHealth-Supported Program for People with TIA. Healthcare. 2021;9(8):968.
28. Association NS. Stroke Risk Score Card 2018 [Available from: https://www.stroke.org/stroke-risk-scorecard-2018/.
29. Cup EH, op Reimer WS, Thijssen MC, van Kuyk-Minis M. Reliability and validity of the Canadian Occupational Performance Measure in stroke patients. Clinical rehabilitation. 2003;17(4):402.
30. Feigin V, Norrving B, Krishnamurthi R, Barker-Collo S, Wang W, Fu HUA, et al. Stroke Riskometer app: A new promising approach for primary stroke prevention and epidemiological research on stroke and other major non-communicable disorders (NCD) across the globe. International Journal of Stroke. 2015;10:101–2.
31. Medvedev O, Truong QC, Merkin A, Borotkanics R, Krishnamurthi R, Feigin V. Cross-cultural validation of the stroke riskometer using generalizability theory. Scientific Reports. 2021;11(1):1–10.
32. Law M, Baptiste S, McColl M, Opzoomer A, Polatajko H, Pollock N. The Canadian occupational performance measure: an outcome measure for occupational therapy. Can J Occup Ther. 1990;57(2):82–7.
33. Fugl-Meyer AR, Melin R, Fugl-Meyer KS. Life satisfaction in 18-to 64-year-old Swedes: in relation to gender, age, partner and immigrant status. Journal of rehabilitation medicine. 2002;34(5):239–46.
34. Burstrom K, Johannesson M, Diderichsen F. Swedish population health-related quality of life results using the EQ-5D. Qual Life Res. 2001;10(7):621-35.
35. Socialstyrelsen. Nationella riktlinjer för prevention och behandling vid ohälsosamma levnadsvanor. 2018.
36. Atler KE. User-perceived utility of the Daily Experiences of Pleasure, Productivity, and Restoration Profile. Can J Occup Ther. 2015;82(4):235–44.
37. Atler KE, Barney L, Moravec A, Sample PL, Fruhauf CA. The Daily Experiences of Pleasure, Productivity, and Restoration Profile: A case study: Etude de cas sur l'utilite du Daily Experiences of Pleasure, Productivity, and Restoration Profile. Can J Occup Ther. 2017;84(4-5):262–72.
38. Dearborn JL, McCullough LD. Perception of risk and knowledge of risk factors in women at high risk for stroke. Stroke. 2009;40(4):1181–6.

For peer review only - http://bmjopen.bmj.com/site/about/guidelines.xhtml

39. Prochaska JO, Redding CA, Evers KE. The transtheoretical model and stages of change. Health behavior: Theory, research, and practice. 2015:125–48.
40. Aguilar-Farías N, Brown WJ, Peeters GG. ActiGraph GT3X+ cut-points for identifying sedentary behaviour in older adults in free-living environments. Journal of science and medicine in sport. 2014;17(3):293–9.
41. Boutron I, Moher D, Altman DG, Schulz KF, Ravaud P. Extending the CONSORT statement to randomized trials of nonpharmacologic treatment: explanation and elaboration. Annals of internal medicine. 2008;148(4):295–309.
42. Chan A-W, Tetzlaff JM, Altman DG, Laupacis A, Gøtzsche PC, Krleža-Jerić K, et al. SPIRIT 2013 Statement: Defining standard protocol items for clinical trials. Ann Intern Med. 2013;158(3):200-207
43. Asaba E, Bergström A, Patomella A-H, Guidetti S. Engaging occupations among persons at risk for stroke: a health paradox. Scandinavian journal of occupational therapy. 2020:1-10.
44. Dahlgren C, Dackehag M, Wändell P, Rehnberg C. Determinants for use of direct-to-consumer telemedicine consultations in primary healthcare—a registry based total population study from Stockholm, Sweden. BMC family practice. 2021;22(1):1–10.
45. Mouton A, Cloes M. Efficacy of a web-based, center-based or combined physical activity intervention among older adults. Health Education Research. 2015;30(3):422–35.

Figure 1: An example of a checklist that shows the domains that the participants need to register in the app. Published with permission from ScientificMed Tech/Cuviva AB.

For peer review only

For peer review only - http://bmjopen.bmj.com/site/about/guidelines.xhtml

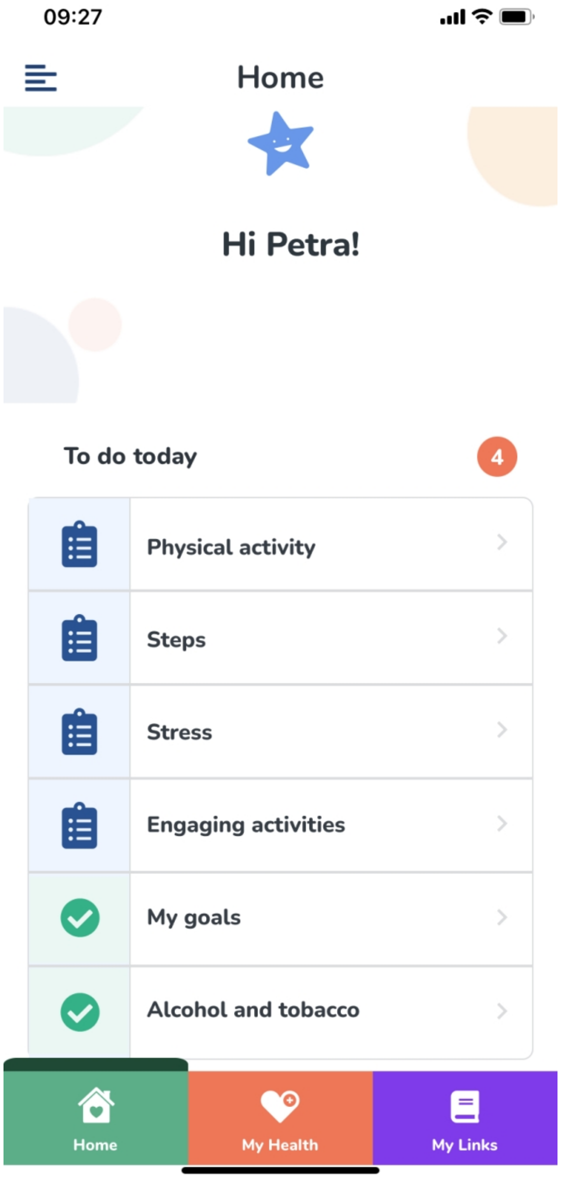

Figure 1: An example of a checklist that shows the domains that the participants need to register in the app. Published with permission from ScientificMed Tech/Cuviva AB.

751x1625mm (28 x 28 DPI)

Reporting checklist for protocol of a clinical trial.

Based on the SPIRIT guidelines.

Instructions to authors

Complete this checklist by entering the page numbers from your manuscript where readers will find each of the items listed below.

Your article may not currently address all the items on the checklist. Please modify your text to include the missing information. If you are certain that an item does not apply, please write "n/a" and provide a short explanation.

Upload your completed checklist as an extra file when you submit to a journal.

In your methods section, say that you used the SPIRITreporting guidelines, and cite them as:

Chan A-W, Tetzlaff JM, Altman DG, Laupacis A, Gøtzsche PC, Krleža-Jerić K, Hróbjartsson A, Mann H, Dickersin K, Berlin J, Doré C, Parulekar W, Summerskill W, Groves T, Schulz K, Sox H, Rockhold FW, Rennie D, Moher D. SPIRIT 2013 Statement: Defining standard protocol items for clinical trials. Ann Intern Med. 2013;158(3):200-207

		Reporting Item	Page Number
**Administrative information**			
Title	#1	Descriptive title identifying the study design, population, interventions, and, if applicable, trial acronym	1

For peer review only

Trial registration	#2a	Trial identifier and registry name. If not yet registered, name of intended registry	2
Trial registration: data set	#2b	All items from the World Health Organization Trial Registration Data Set	N/A a registration has not been done
Protocol version	#3	Date and version identifier	2
Funding	#4	Sources and types of financial, material, and other support	20
Roles and responsibilities: contributorship	#5a	Names, affiliations, and roles of protocol contributors	20
Roles and responsibilities: sponsor contact information	#5b	Name and contact information for the trial sponsor	N/A, no trial sponsor
Roles and responsibilities: sponsor and funder	#5c	Role of study sponsor and funders, if any, in study design; collection, management, analysis, and interpretation of data; writing of the report; and the decision to submit the report for publication, including whether they will have ultimate authority over any of these activities	N/A

For peer review only

Roles and responsibilities: committees	#5d	Composition, roles, and responsibilities of the coordinating centre, steering committee, endpoint adjudication committee, data management team, and other individuals or groups overseeing the trial, if applicable (see Item 21a for data monitoring committee)	N/A
**Introduction**			
Background and rationale	#6a	Description of research question and justification for undertaking the trial, including summary of relevant studies (published and unpublished) examining benefits and harms for each intervention	7-9
Background and rationale: choice of comparators	#6b	Explanation for choice of comparators	8-9
Objectives	#7	Specific objectives or hypotheses	9
Trial design	#8	Description of trial design including type of trial (eg, parallel group, crossover, factorial, single group), allocation ratio, and framework (eg, superiority, equivalence, non-inferiority, exploratory)	9
**Methods: Participants,**			

For peer review only

**interventions, and outcomes**			
Study setting	#9	Description of study settings (eg, community clinic, academic hospital) and list of countries where data will be collected. Reference to where list of study sites can be obtained	9
Eligibility criteria	#10	Inclusion and exclusion criteria for participants. If applicable, eligibility criteria for study centres and individuals who will perform the interventions (eg, surgeons, psychotherapists)	10-11
Interventions: description	#11a	Interventions for each group with sufficient detail to allow replication, including how and when they will be administered	11-12
Interventions: modifications	#11b	Criteria for discontinuing or modifying allocated interventions for a given trial participant (eg, drug dose change in response to harms, participant request, or improving / worsening disease)	N/A
Interventions: adherance	#11c	Strategies to improve adherence to intervention protocols, and any procedures for monitoring adherence (eg, drug tablet return; laboratory tests)	N/A
Interventions: concomitant care	#11d	Relevant concomitant care and interventions that are permitted or prohibited during the trial	N/A

For peer review only

Outcomes	#12	Primary, secondary, and other outcomes, including the specific measurement variable (eg, systolic blood pressure), analysis metric (eg, change from baseline, final value, time to event), method of aggregation (eg, median, proportion), and time point for each outcome. Explanation of the clinical relevance of chosen efficacy and harm outcomes is strongly recommended	13-14
Participant timeline	#13	Time schedule of enrolment, interventions (including any run-ins and washouts), assessments, and visits for participants. A schematic diagram is highly recommended (see Figure)	15
Sample size	#14	Estimated number of participants needed to achieve study objectives and how it was determined, including clinical and statistical assumptions supporting any sample size calculations	10
Recruitment	#15	Strategies for achieving adequate participant enrolment to reach target sample size	10

Methods: Assignment of interventions (for controlled trials)

For peer review only

Allocation: sequence generation	#16a	Method of generating the allocation sequence (eg, computer-generated random numbers), and list of any factors for stratification. To reduce predictability of a random sequence, details of any planned restriction (eg, blocking) should be provided in a separate document that is unavailable to those who enrol participants or assign interventions	10-11
Allocation concealment mechanism	#16b	Mechanism of implementing the allocation sequence (eg, central telephone; sequentially numbered, opaque, sealed envelopes), describing any steps to conceal the sequence until interventions are assigned	11
Allocation: implementation	#16c	Who will generate the allocation sequence, who will enrol participants, and who will assign participants to interventions	N/A not been decided
Blinding (masking)	#17a	Who will be blinded after assignment to interventions (eg, trial participants, care providers, outcome assessors, data analysts), and how	10
Blinding (masking): emergency unblinding	#17b	If blinded, circumstances under which unblinding is permissible, and procedure for revealing a participant's allocated intervention during the trial	N/A

For peer review only

**Methods: Data collection, management, and analysis**			
Data collection plan	#18a	Plans for assessment and collection of outcome, baseline, and other trial data, including any related processes to promote data quality (eg, duplicate 6measurements, training of assessors) and a description of study instruments (eg, questionnaires, laboratory tests) along with their reliability and validity, if known. Reference to where data collection forms can be found, if not in the protocol	13-15
Data collection plan: retention	#18b	Plans to promote participant retention and complete follow-up, including list of any outcome data to be collected for participants who discontinue or deviate from intervention protocols	N/A not been decided
Data management	#19	Plans for data entry, coding, security, and storage, including any related processes to promote data quality (eg, double data entry; range checks for data values). Reference to where details of data management procedures can be found, if not in the protocol	15

For peer review only

Statistics: outcomes	#20a	Statistical methods for analysing primary and secondary outcomes. Reference to where other details of the statistical analysis plan can be found, if not in the protocol	17-18
Statistics: additional analyses	#20b	Methods for any additional analyses (eg, subgroup and adjusted analyses)	N/A
Statistics: analysis population and missing data	#20c	Definition of analysis population relating to protocol non-adherence (eg, as randomised analysis), and any statistical methods to handle missing data (eg, multiple imputation)	Not decided
**Methods: Monitoring**			
Data monitoring: formal committee	#21a	Composition of data monitoring committee (DMC); summary of its role and reporting structure; statement of whether it is independent from the sponsor and competing interests; and reference to where further details about its charter can be found, if not in the protocol. Alternatively, an explanation of why a DMC is not needed	N/A
Data monitoring: interim analysis	#21b	Description of any interim analyses and stopping guidelines, including who will have access to these interim results and make the final decision to terminate the trial	N/A

For peer review only

Harms	#22	Plans for collecting, assessing, reporting, and managing solicited and spontaneously reported adverse events and other unintended effects of trial interventions or trial conduct	N/A
Auditing	#23	Frequency and procedures for auditing trial conduct, if any, and whether the process will be independent from investigators and the sponsor	N/A
**Ethics and dissemination**			
Research ethics approval	#24	Plans for seeking research ethics committee / institutional review board (REC / IRB) approval	19
Protocol amendments	#25	Plans for communicating important protocol modifications (eg, changes to eligibility criteria, outcomes, analyses) to relevant parties (eg, investigators, REC / IRBs, trial participants, trial registries, journals, regulators)	N/A
Consent or assent	#26a	Who will obtain informed consent or assent from potential trial participants or authorised surrogates, and how (see Item 32)	11
Consent or assent: ancillary studies	#26b	Additional consent provisions for collection and use of participant data and biological specimens in ancillary studies, if applicable	N/A

For peer review only

Confidentiality	#27	How personal information about potential and enrolled participants will be collected, shared, and maintained in order to protect confidentiality before, during, and after the trial	19
Declaration of interests	#28	Financial and other competing interests for principal investigators for the overall trial and each study site	20
Data access	#29	Statement of who will have access to the final trial dataset, and disclosure of contractual agreements that limit such access for investigators	19, has been informed to the ethics review board
Ancillary and post trial care	#30	Provisions, if any, for ancillary and post-trial care, and for compensation to those who suffer harm from trial participation	None
Dissemination policy: trial results	#31a	Plans for investigators and sponsor to communicate trial results to participants, healthcare professionals, the public, and other relevant groups (eg, via publication, reporting in results databases, or other data sharing arrangements), including any publication restrictions	20
Dissemination policy: authorship	#31b	Authorship eligibility guidelines and any intended use of professional writers	20

For peer review only

Dissemination policy: reproducible research	#31c	Plans, if any, for granting public access to the full protocol, participant-level dataset, and statistical code	None

Appendices

Informed consent materials	#32	Model consent form and other related documentation given to participants and authorised surrogates	N/A the study was granted including consent forms, by national review board
Biological specimens	#33	Plans for collection, laboratory evaluation, and storage of biological specimens for genetic or molecular analysis in the current trial and for future use in ancillary studies, if applicable	N/A

None The SPIRIT checklist is distributed under the terms of the Creative Commons Attribution License CC-BY-ND 3.0. This checklist can be completed online using https://www.goodreports.org/, a tool made by the EQUATOR Network in collaboration with Penelope.ai

For peer review only

BMJ Open

Make My Day: Primary prevention of stroke using engaging everyday activities as a mediator of sustainable health—a randomised controlled trial and process evaluation protocol

Journal:	*BMJ Open*
Manuscript ID	bmjopen-2023-072037.R1
Article Type:	Protocol
Date Submitted by the Author:	04-Jul-2023
Complete List of Authors:	Patomella, Ann-Helen; Karolinska Institutet, Department of Neurobiology, Care Sciences and Society; Academic Primary Health Care Centre<br>Guidetti, Susanne; Karolinska Institutet, Department of Neurobiology, Care Sciences and Society; Karolinska University Hospital<br>Hagströmer, Maria; Karolinska Institute, Department of Neurobiology Care Sciences and society; Academic Primary Health Care Centre<br>Olsson, Christina; Academic Primary Health Care Centre, ; Karolinska Institutet, Department of Neurobiology, Care Sciences and Society<br>Jakobsson, E; Karolinska Institutet, Department of Neurobiology, Care Sciences and Society<br>Nilsson, Gunnar; Academic Primary Health Care Centre; Karolinska Institutet, Department of Neurobiology, Care Sciences and Society<br>Åkesson, Elisabet; Karolinska Institutet, Department of Neurobiology, Care Sciences and Society; Stockholms Sjukhem FoUU<br>Asaba, Eric; Karolinska Institutet, Department of Neurobiology, Care Sciences and Society; Stockholms Sjukhem FoUU
**Primary Subject Heading**:	Health services research
Secondary Subject Heading:	Cardiovascular medicine, Neurology, Rehabilitation medicine
Keywords:	Stroke < NEUROLOGY, PREVENTIVE MEDICINE, REHABILITATION MEDICINE

SCHOLARONE™
Manuscripts

Make My Day: Primary prevention of stroke using engaging everyday activities as a mediator of sustainable health—a randomised controlled trial and process evaluation protocol

Patomella Ann-Helen, ann-helen.patomella@ki.se, (ORCID 0000-0003-2667-4073)[1, 2]

Guidetti Susanne, Susanne.guidetti@ki.se, (ORCID 0000-0001-6878-6394) [1,3]

Hagströmer Maria, maria.hagstromer@ki.se, (ORCID 0000-0002-4607-8677) [1, 2]

Olsson Christina B, christina.b.olsson@regionstockholm.se, (0000-0003-1300-7765) [2,4]

Jakobsson Elin, elin.jakobsson@ki.se, (ORCID 0000-0001-9756-1354) [1]

Nilsson Gunnar, gunnar.nilsson@ki.se, (ORCID 0000-0001-7811-7602) [2,5]

Åkesson Elisabet, elisabet.akesson@ki.se, (ORCID 0000-0002-8227-9118) [6,7]

Asaba Eric, eric.asaba@ki.se, (ORCID 0000-0002-6910-3468)[1,7]

Affiliation:

[1]Department of Neurobiology, Care Sciences and Society, Division of Occupational Therapy, Karolinska Institutet, Stockholm, Sweden

[2] Academic Primary Health Care Centre, Region Stockholm, Stockholm, Sweden

[3] Women´s Health and Allied Health Professionals Theme, Medical Unit Occupational therapy and Physiotherapy, Karolinska University Hospital

[4] Department of Neurobiology, Care Sciences and Society, Division of Physiotherapy, Karolinska Institutet, Stockholm, Sweden

[5] Department of Neurobiology, Care Sciences and Society, Division of Family Medicine and Primary Care, Karolinska Institutet, Stockholm, Sweden

[6] Department of Neurobiology, Care Sciences and Society, Division of Neurogeriatrics, Karolinska Institutet, Stockholm, Sweden

[7] R&D Unit, Stockholms Sjukhem, Stockholm, Sweden

For peer review only - http://bmjopen.bmj.com/site/about/guidelines.xhtml

Corresponding author:
Ann-Helen Patomella, Department of Neurobiology, Care Sciences and Society, Division of Occupational Therapy, Karolinska Institutet, Stockholm, Sweden
Email address: ann-helen.patomella@ki.se
Phone number: +46 852483726

* I, the Submitting Author has the right to grant and does grant on behalf of all authors of the Work (as defined in the below author licence), an exclusive licence and/or a non-exclusive licence for contributions from authors who are: i) UK Crown employees; ii) where BMJ has agreed a CC-BY licence shall apply, and/or iii) in accordance with the terms applicable for US Federal Government officers or employees acting as part of their official duties; on a worldwide, perpetual, irrevocable, royalty-free basis to BMJ Publishing Group Ltd ("BMJ") its licensees and where the relevant Journal is co-owned by BMJ to the co-owners of the Journal, to publish the Work in BMJ Open and any other BMJ products and to exploit all rights, as set out in our licence.

The Submitting Author accepts and understands that any supply made under these terms is made by BMJ to the Submitting Author unless you are acting as an employee on behalf of your employer or a postgraduate student of an affiliated institution which is paying any applicable article publishing charge ("APC") for Open Access articles. Where the Submitting Author wishes to make the Work available on an Open Access basis (and intends to pay the relevant APC), the terms of reuse of such Open Access shall be governed by a Creative Commons licence – details of these licences and which Creative Commons licence will apply to this Work are set out in our licence referred to above.

Word count: 3317
Protocol version: 29th of June 2023. Version 2.0
Trial Registration number: ClinicalTrials.gov Identifier: NCT05279508

For peer review only - http://bmjopen.bmj.com/site/about/guidelines.xhtml

Abstract

Introduction

The individual, societal and economic benefits of stroke prevention are high. Even though most risk factors can be reduced by changes to lifestyle habits, maintaining new and healthy activity patterns has been shown to be challenging.

The aim of the study is to evaluate the impact of an interdisciplinary team-based, mHealth-supported prevention intervention on persons at risk for stroke. The intervention is mediated by engaging everyday activities that promote health. An additional aim is to describe a process evaluation that serves to increase knowledge about how the program leads to potential change by studying the implementation process and mechanisms of impact.

Methods and analysis

The study will be a randomised controlled trial including 104 persons at risk for stroke. Persons at risk of stroke (n =52) will be randomised to an mHealth-supported stroke prevention program. Controls will have ordinary Primary healthcare (PHC) services. The 10-week programme will be conducted at PHC clinics, combining group meetings and online resources to support self-management of lifestyle change using engaging everyday activities as a mediator. Primary outcomes are stroke risk, lifestyle habits, and participation in health-promoting activities. Assessments will be performed at baseline and at follow-up (11 weeks and 12 months). Effects of the programme will be analysed using inferential statistics. Implementation will be analysed using qualitative and quantitative methods.

Ethics and dissemination

The study has been approved by the Swedish Ethical Review Authority. Study results will be disseminated in peer-reviewed journals and at regional and international conferences targeting mixed audiences.

For peer review only

For peer review only - http://bmjopen.bmj.com/site/about/guidelines.xhtml

Strengths and limitations of this study

- A robust randomised controlled trial (RCT) design will be used to investigate the effectiveness of the intervention programme.
- A process evaluation will provide rich data, useful for analysing the outcomes and the research process
- A limitation is that primary outcomes are based on self-reported data.
- An inclusion criterion is motivation for change which can skew the results and lower external validity.

For peer review only

For peer review only - http://bmjopen.bmj.com/site/about/guidelines.xhtml

INTRODUCTION

In Sweden, a health care reform programme entitled "Good Quality Local Health Care—Primary Care Reform" (1) is currently paving the way for a large-scale transformation. The reform targets primary health care (PHC) specifically, and is designed to proactively address and prevent illness at an early stage to reduce the burden of non-communicable diseases (NCD), like stroke. The reform is in line with the United Nations Sustainable Development Goals, urging governments to reduce premature mortality from NCDs by 1/3 by year 2030 through prevention and treatment (2). By the age of 55, the risk for stroke increases considerably and doubles each decade afterward (3,4). Although the incidence of stroke has decreased in the general population, trends show an increase in stroke incidence in young adults (5). In Sweden, stroke incidence has declined by over 40% the last 15 years (6). However, both globally and in Sweden, cerebrovascular diseases like stroke continue to be the most common cause of death and impairment (7).

The individual, societal, and economic benefits of stroke prevention are high. Many of the stroke risk factors are largely addressable; e.g., smoking, obesity, type 2 diabetes, hypertension, physical inactivity, and dietary intake. The benefits of a healthy lifestyle are clear (4,8), however, the long-term effect (follow-up at 12 months or longer) of lifestyle interventions are not (9,10). For example, the effectiveness of PHC-based physical activity interventions is inconclusive (11). There is evidence for short-term improvements, but there is a lack of evidence for long-term effects (9). A multi-factorial approach to stroke prevention is warranted. A systematic review showed that multifactorial lifestyle habit interventions have greater potential effect on reducing risk factors than single-factor interventions (12). Preliminary evidence exists from a Swedish trial on a multifactorial lifestyle counselling program (13) that improved physical activity and dietary habits and reduced smoking and stress; however, the study was not conducted in PHC and did not have a control group. mHealth (a term for the combination of eHealth services and smartphone technology (14)) presents possibilities for accessibility and affordability when developing health services (15,
16) making it an excellent tool for designing stroke prevention models (17).

Theoretical concept of the intervention program

For peer review only - http://bmjopen.bmj.com/site/about/guidelines.xhtml

The program is a complex intervention, and the Medical Research Council (MRC) guidance for developing and evaluating complex interventions (18) has been utilised in the design of the study. MRC suggest several key elements and stages; Development, Feasibility/piloting, Evaluation, and Implementation. This randomised controlled trial (RCT) evaluates the effectiveness and implementation of the Make My Day (MMD) programme and is based on already-completed or ongoing studies concerning development and feasibility.

Engaging everyday activity

In the MMD programme, engaging everyday activities (EEA) are seen as the means and goal for changing and sustaining a healthy lifestyle in the intervention program, and while it is not a new concept, it has not been studied in relation to changing lifestyle habits to prevent NCDs. EEA is defined as a special type of activity that: is based on individual experience, filled with meaning, and gives a sense of intense participation and enthusiasm to the individual (19, 20). Examples from previous studies show that EEA can include a variety of activities such as working, playing computer games, and reading books. We recently showed that an intervention programme targeting cardiovascular disease prevention could benefit from incorporating health promoting EEA (21). The complexity of changing lifestyle habits has been described as a paradox between EEA and health (22). Even though EEA is subjectively meaningful and engaging to an individual, it might have an arguably negative impact on health; for example, engagement in sedentary activity or in unhealthy behaviours (22). In the current project, EEAs are seen as having the potential to change everyday activity patterns, and when carefully designed (for example, listening to an audiobook while taking a walk), incorporate a routine that promotes sustainable health among persons at risk for stroke. Studies have shown that promoting EEAs can have positive health impacts for older adults (23–25). Studies on populations that live lives that incorporate EEAs combined with moderate-intensity physical activities and a healthy diet indicate a strong relation to wellbeing, longevity, and cultural context (4,5).

Current state-of-the art in stroke prevention suggest the need for a multifactorial PHC intervention that address modifiable risk factors for stroke based on individual needs

For peer review only - http://bmjopen.bmj.com/site/about/guidelines.xhtml

and engagement in everyday activities that promote health with the support of a mHealth service.

Objectives of the proposed study

The main aim of the study is to evaluate the impact of an inter-disciplinary team-based, mHealth-supported prevention intervention in primary health care (PHC) —mediated with engaging everyday activities that promote health—to decrease stroke risk (primary outcome), and increase participation in engaging everyday activities and Health Related Quality of Life (HRQoL). The aim is also to describe a process evaluation that serves to increase knowledge about how the program leads to potential change by studying the implementation process and mechanisms of impact.

Hypothesis

We hypothesise that the Make My Day (MMD) intervention programme is more beneficial than regular PHC services (control group) in decreasing stroke risk (primary outcome). We also hypothesised that MMD is more beneficial than regular PHC in increasing a) participation in health promoting EEAs, and b) HRQoL.

METHODS AND ANALYSIS

Design

The study will be a randomised, assessor-blinded, controlled trial of persons at risk for stroke. The process evaluation answers questions as to what interventions were delivered and how by combining qualitative interviews and descriptive quantitative data on the implementation, casual mechanisms, and contextual variation (26).

Study setting

The study will be conducted in close collaboration with four PHC clinics in the Stockholm area (different parts of Stockholm to represent a diversity in geographical area). PHC clinics in this study are rehabilitation units involving dietitians, physiotherapists, and occupational therapists. In Region Stockholm, rehabilitation units are often both organisationally and geographically separate from general practitioner (GP) primary health care clinics. These primary health care rehabilitation units have an agreement with the County Council in Stockholm, and are available for patients to choose from without the need of a referral for treatment by certified

For peer review only - http://bmjopen.bmj.com/site/about/guidelines.xhtml

physiotherapists, occupational therapists, and dieticians. PHC services are publicly funded in Sweden.

Sample size and power considerations

The primary outcome is stroke risk, with emphasis on modifiable stroke risk factors. Based on data from a case study (27), we calculate a decrease of at least one level of stroke risk (e.g., moving from high risk to medium risk in the Stroke Risk Score Card (28)) with a standard deviation of 1.5, statistical power of 80%, with two-railed α =0.05. Under these assumptions, the required sample size was 35 in each group (in total n=70). The Stroke Risk Score Card was developed as an easy-to-use self-assessment tool by the National Stroke Association in the United Kingdom (28). The tool has been used previously in a few studies to detect risk factors of stroke (27,28) and in a recently-finished pilot study conducted in the research group (publication in manuscript). Since the Stroke Risk Score Card has not been sufficiently tested psychometrically, power calculations were added for performance in EEAs. Assuming a difference on performance in EEAs of two points (as measured with the Canadian Occupational Performance Measure, COPM (29)) a power of 0.8 and a two-sided p value of 0.05 a sample size of 40 participants in each group would be sufficient.

To safeguard against dropouts (a maximum 30% dropout rate is assumed), a total of 104 participants will be enrolled in the study (52 in each group).

Participants: Recruitment and eligibility criteria

Persons at risk of stroke will be included in the project and participants will be recruited via advertisements in social media, a webpage, and flyers at PHCs. A stroke risk online screening survey will be used to find eligible participants. Inclusion criteria for the study are a) three or more risk factors deemed ‘high risk’ using the Stroke Risk Score Card, b) motivation for lifestyle change and to participate in a digital lifestyle intervention (including use of a smart phone), d) aged between 55–75 years old, and without a diagnosis of dementia or cognitive impairment hindering participation. Exclusion criteria include having previously had a stroke or a trans-ischemic attack diagnosis, lack of understanding of the Swedish language, and not being able to use a mobile phone application. A total sample of n =104 participants (persons at risk of stroke), divided into two arms (52+52) for intervention and controls

For peer review only - http://bmjopen.bmj.com/site/about/guidelines.xhtml

is estimated. Block randomisation will be utilised with a block size of four (25). Allocation will be done following baseline assessment by a researcher not involved in data collection nor intervention. The assessors of outcomes will be blinded to allocation until the end of the study.

In addition, next of kin to persons at risk for stroke in the intervention group are also invited to answer questions (survey and interview) regarding support of their relative. Persons at risk for stroke who do not have a next of kin will not be excluded from the study. PHC professionals that have been trained and delivered the interventions programme will be additional participants in the process evaluation of the study. Stakeholders (such as leaders at the involved PHC clinics) will be invited to individual interviews.

Informed consent

Written informed consent will be obtained from all participants (persons at risk, their next of kin, and PHC staff and stakeholders) at the start of recruitment. Information about the study will be given in written and verbal forms during meetings with research staff. Persons at risk will be asked to identify a next of kin that will be asked to participate by the researchers.

Make My Day—a stroke-prevention program

The MMD intervention program enables healthy activity patterns and aims to reduce the risk of stroke by means of four strategies: a) the incorporation of health-promoting EEAs, b) the use of mobile phone technology (mHealth) to increase health literacy, and awareness of current habits and fostering self-management, c) setting realistic goals that form new habits that prompt conscious decisions for healthy choices and habits, and d) sharing experience in a learning environment.

Duration and specific content of the intervention programme

The MMD stroke intervention programme is a 10-week group programme consisting of five sessions over the first five weeks, followed by a sixth booster session five weeks later. During the intervention, participants will work actively on self-chosen goals, EEAs, and habits to change behaviour and lifestyle. A mobile phone app will be used by participants throughout the 10 weeks, supporting their awareness of

For peer review only - http://bmjopen.bmj.com/site/about/guidelines.xhtml

current lifestyle habits and everyday activities. To form new habits, common situations will be used to cue behaviour change, like seeing an elevator and looking for the staircase, prompting health-promoting behaviour, and making a conscious decision to walk the stairs (30). The continuation of a change process is expected from the participants following the 10-week program period, and strategies for self-management are anticipated.

Each session (90 min) has a theme and includes some type of activity such as exercise, making a light snack, or taking a walk. Group dynamics and personal experience are used to reflect on EEAs in relation to health, doing and future goals. The sessions and content, presented in Table 1, are delivered by a trained health professional, for example an occupational therapist, physiotherapist, or dietician. There are sessions week 1-5 and week 10. During week 6-9, no sessions are held, instead the participants are expected to self-manage these weeks with the support of a mobile phone app.

The health care professionals who will provide the MMD program/intervention will participate in structured education specially designed for this program. This education will be given in an on-site and digital combination on three occasions, and will be held by two research team members with extensive experience in pedagogy and in the research protocol. In addition, the health professionals will have access to a digital educational platform with rich and varied material, and all material to be used during the 10-week programme. To avoid contamination, the health professionals are instructed to not deliver the 10-week program to other patients during the research period.

Table 1: Summary of session themes, concepts, and activities supporting a change process.

Week	Session theme	Profession	Concepts	Activity
1	**1:** Risk factors for stroke and engaging everyday activities	Occupational therapist	Health literacy concerning stroke risk, engaging activities, change process, expectations	Peer interview on engaging activities. Learn how to register in the app
2	**2:** Physical activity	Physiotherapist	Physical activity, physical inactivity	Try a physical group exercise class at a gym

For peer review only - http://bmjopen.bmj.com/site/about/guidelines.xhtml

3	**3:** Diet and health	Dietician	Dietary routines and change	Food lab—prepare and test; e.g., healthy snacks/sandwiches
4	**4:** Balanced everyday life	Occupational therapist	Activity balance and stressors	Relaxation—such as medical yoga or meditation
5	**5:** The meaning of healthy habits, routines, and activity patterns	One of the team members	Current and desired routines/habits, activity patterns and resources	Walk-and-talk—e.g., in a forest or a historical walk in the city
10	**6:** Booster session: Evaluation and the road ahead	Occupational therapist	Self-management, sustainability, view of the self, social support, revisiting goals/new goals, and social aspects of health	Preparing healthy snacks and group reflection on the program

The mobile phone app

The app for the pilot project was produced by collaboration with ScientificMed Tech AB (now part of Cuviva AB) (http://www.scientificmed.com), and for the current project the app has been modified based on previous experiences of the users and the researchers. A workshop with the pilot study participants, researchers and the company showed that the participants wanted the app to be more tailored to their needs with a more user-friendly interface. Both the researchers and the users wanted feedback to be relevant and tailored to the users progress thus supporting change and awareness. As with the previous version, the new version of the app includes six domains for registering daily activities, experiences, and behaviours (see Figure 1 for examples from the app): my goals (goal achievements on three pre-set goals); physical activity and steps (step counts, 24-hour time use in relation to exercise, moderately intense activities, sleep, sedentary activities, and other activities); engaging activities (participating in health-promoting EEAs); tobacco and alcohol use (consumption); stress (perceived time-pressure); and dietary habits (consumption of fruits/vegetables, breakfast, fish and snacks—not included in the figure). Domains are based on modifiable risk factors for stroke, as presented by the American Heart Association (3), with the addition of health-promoting EEAs and stress reduction.

-Insert Figure 1 here-

Data collection

Persons at risk for stroke

Data collection with a research assistant starts with an individual meeting (baseline) with all eligible participants, just before the meeting (baseline minus 2 days), during

For peer review only - http://bmjopen.bmj.com/site/about/guidelines.xhtml

which participants are sent a link to an online survey for collecting self-reported measures. During baseline assessment, all participants (including controls) will be informed of their stroke risk factors. Motivational interviewing techniques will be used to identify three problem areas in relation to lifestyle habits and stroke risk factors, and these areas will be used to formulate three lifestyle change goals. Allocation (randomisation) will be done following baseline assessment. Allocation sequences will be done by an independent researcher not involved in data collection or intervention. The researchers who are assessors of outcomes will be blinded to allocation until the end of the study. The assessments measuring primary and secondary outcomes will be collected at baseline, at follow-up (11 weeks), and at 12 months; see Table 2. Demographic data will be collected at baseline. Process data will be collected continuously. Controls will be offered standard care by PHCs as needed during the 12 months study period.

Outcome data

Outcome assessment methods were carefully chosen to assure methods that are valid and reliable and will capture change. The primary outcome measure is risk for stroke, measured by the Swedish version of the *Stroke Riskometer* (30,31) ~~and the Stroke Risk Score Card (27, 28)~~. Secondary outcomes include participation in health-promoting everyday activities, measured by the *Canadian Occupational Performance Measure*, (COPM) (32), and self-rated health measured using *LiSat-11* (33) and *EQ-5D* (34). Other measures are lifestyle habits (measured using the updated *Swedish Lifestyle Survey*, Levnadsvaneenkäten) (35), and activity patterns, as measured using the Swedish version of the *Productivity Pleasure and Restoration Profile* (36, 37). Survey data will be gathered for health literacy of stroke risk (38), experiences of time pressure (stress), cost effectiveness (e.g., self-reported sick leave; health care utilisation, and use of medication), readiness and motivation for change (39), current mobile phone use, and mapping out engaging everyday activities. Habitual physical activity will be measured using the activPAL® micro activity monitor (PAL Technologies Ltd., Glasgow, UK) (40). The activPAL is a small device which provides information on position and acceleration of the body. The monitor is attached to the thigh and will be worn for five consecutive days after baseline and at follow-up (11 weeks and 12 months). Outcomes from the monitor are 1) time spent sitting/lying, standing, stepping, 2) numbers of step counts, and 3) sit-to-stand transitions.

For peer review only - http://bmjopen.bmj.com/site/about/guidelines.xhtml

Outcome data- next of kin

We will collect data from next of kins to the participants in the intervention group via an online survey. The survey will include demographic measures of health and questions on their view of the program and the support they have given their kin during the intervention period.

Process data

The process evaluation will illuminate casual mechanisms and help identify factors that are associated with variation in outcomes, such as contextual and external factors (26). Process data include both qualitative and quantitative descriptive data, including logbooks from PHC staff (notes taken during delivery of the program), course evaluations from the web-based staff training, and semi-structured exit interviews with participants at risk for stroke and their next of kin, see Table 2. Fidelity will be evaluated as the extent to which the programme was delivered as expected. Dose will be assessed as the quantity of the implemented intervention. Adaptation, such as changes made to fit different PHC settings, will be collected during interviews with PHC staff. Reach will be assessed regarding how many eligible patients signed up and how many completed the MMD program. In addition, adverse events will be registered. Context includes external factors that may act as a barrier or facilitator to the implementation itself and to the interventions' effects. Assessing barriers and facilitators to program implementation will also involve evaluating program feasibility; i.e., the extent to which stakeholders regard the MMD as satisfactory in terms of content and complexity/difficulty.

Data will be managed using an online software called RedCap (https://www.project-redcap.org/) in combination with a local data management system.

Participant timeline

Participant enrollment started in April 2022, and in June 2023 all 104 participants had been included. The last groups' 12-month follow-up will occur in March 2024 (marking the end of the study). In total, five intervention groups, each consisting of 10–12 participants will receive the prevention program during the study.

For peer review only - http://bmjopen.bmj.com/site/about/guidelines.xhtml

Table 2: Summary of measures to be collected

	Instrument and scale	Time points
**Primary outcome measures from persons at risk for stroke:**		
Stroke risk	The Stroke Riskometer*, the Stroke risk score card*	t1, t2, t3
**Secondary outcome measures:**		
Participation in everyday activities	COPM, PPR profile*	t1, t2, t3
Physical activity (habitual)	ActivPal	t1, t2, t3
Life satisfaction	EQ-5D*, LiSat-11*	t1, t2, t3
Lifestyle habits	The Swedish Lifestyle Habits Questionnaire*	t1, t2, t3
**Demographics and measures:**		
Age	Year*	t1
Gender	Male/Female/Other*	t1
Ethnic background	Mother tongue*, place of birth*	t1
Height	Cm	t1
Weight	Kg*	t1, t2, t3
Living situation	Living alone or not*	t1, t3
Yearly income	In Swedish crona*	t1, t3
Employment status	Part-time, full-time, sick-leave, unemployed, student, retired*	t1, t3
Level of education	Years of education*	t1
Blood pressure	mmHg	t1, t2, t3
Health literacy	Knowledge of stroke	t1, t2, t3
Motivation for change	Self-reported, ordinal scale	t1
Cost effectiveness	Self-reported sick-leave and absence from work past six months; health care utilisation past six months; Use of medication	t1, t2, t3
**Experiences of next of kin:**	Self-reported health and support*	t2, t3
**Process data:**		

For peer review only - http://bmjopen.bmj.com/site/about/guidelines.xhtml

Fidelity and adaptations	Interviews with interventionists on delivery of intervention. Log-books from interventionists.	t2
Dose	Log-books from interventionists.	t2

t1 =Baseline; t2 =One week following intervention ending; t3 =12 months follow-up post baseline
* = measures collected via an online survey

Data Analysis Plan

Outcomes on effects

The characteristics of all persons at risk for stroke at inclusion, and outcomes at 11 weeks and 12 months after inclusion, will be presented with descriptive statistics. The treatment effects in the RCT study will be analysed on an intention-to-treat basis, with randomised participants retaining their original allocated group, and measured as differences between groups at follow-up and at 12 months considering plausible confounders. Outcome data will be examined for outliers, normality, and missing data. Analyses of covariance will be used for continuous outcomes, with baseline values as covariates. Logistic regression analyses will be used for dichotomous outcomes. The level of significance will be set at $p \leq 0.05$ and the confidence level at 95%. We will use SPSS to analyse the data. Analyses will provide results for the relative effectiveness of the intervention program. The results will be reported accordance to CONSORT recommendations for reporting randomised controlled trials on non-pharmacological treatments (41) and the protocol has been reported according to the SPIRIT reporting guidelines (42).

Process evaluation

A mixed method approach where qualitative and quantitative data is integrated will be used to answer how the implementation process and potential mechanisms of impact can explain the outcomes of the MMD intervention.

Data collected from surveys, logbooks on recruitment and dropout, and logs from the app registrations will be entered, analysed, and summarised. Descriptive statistical analyses will be conducted to report on the study’s feasibility: recruitment, drop-outs,

For peer review only - http://bmjopen.bmj.com/site/about/guidelines.xhtml

retention rate, and adherence. Data from app registrations will be used to report on feasibility and usability. Qualitative interviews will be transcribed verbatim and analysed using thematic qualitative analyses.

Patient and Public Involvement

The experiences and input from persons at risk for stroke participating in previous studies on the feasibility of the Make My Day programme have informed the development of research questions, materials, and research processes for the current study. The process evaluation will assess the participants burden of the intervention and time required to participate in the research. We plan to disseminate the study results to all participants in a Swedish report and to ask the participants to comment on the report.

Discussion

Several NCDs share the same risk factors as stroke, and an intervention program has the potential to address other NCDs and health in general and should overlap with other health-promoting strategies (3). In the proposed study we will evaluate the MMD program in regard to decreasing stroke risk by using broad strategies and addressing multiple factors of relevance. The theoretical base of the protocol is grounded on EEAs as the mediator and goal for decreasing stroke risk and sustaining personally-relevant healthy living habits. It is important to note that the concept of personal relevance can mean that in a total week of different activities, some engaging activities can potentially be considered unhealthy (i.e., unhealthy eating activity), but the overall pattern of participation in EEA could be designed to include health-promoting EEA as this study promotes. The paradoxicality of EEAs is that the feeling of being engaged can be just as important for health and wellbeing as being physically active (43). Living habits thus needs to be seen as part of a broader life context, in which health and EEAs are continuously renegotiated and thus need to be regularly reassessed within the context of each person’s life situation.

During the COVID-19 pandemic there was a strong increase in online primary health care consultations in Sweden, especially for younger patients with high economical and educational backgrounds who were born in Sweden; meanwhile the older population sought less care and preferred face-to-face consultations (44). Although there is a possibility to deliver the MMD intervention programme completely online

For peer review only - http://bmjopen.bmj.com/site/about/guidelines.xhtml

(no physical meetings), we have decided to run the programme meetings face-to-face. During the 12-month follow-up of the pilot study, which occurred at the end of 2020, participants rated physical meet-ups (the possibility of exchanging experiences with other at-risk persons and group leaders) as highly valued; which is in line with previous studies that showed that a blended intervention approach can be efficient compared to only online or on-site intervention (45). However, whether multiple face-to-face consultations (doses) would be the most efficient is not clear, and is one of the questions for the process evaluation.

The possible limitation of the study will be the reliability of self-reported measures, and there is a risk of bias since reporting might not be accurate, therefore measures such as activPAL, BMI, and blood pressure are complementing the assessments. Although we have planned for five days of activPAL wear (the recommendation is seven days), participants will wear these 24/7, and we will monitor data loss. External validity of the outcomes could be flawed, due to a recruitment process mainly benefiting highly-motivated persons at risk of stroke, and the risk of dropouts in less-motivated participants. The power calculations are based on a stroke risk score that has to our knowledge not been used for power calculations previously nor in intervention studies. However, this is the score used in our previous pilot study and most relevant to use, since the aim of the study focus on modifiable risk factors which is covered in the score. In addition, we have added a power calculation on a secondary outcome.

Ethical dilemmas include that controls are not being supported in the same way as the intervention group and that the recruitment methods could be skewed and fail to reach out to vulnerable groups in society (lower SES) at risk for stroke. The strength of the study lies in the robustness of the RCT design, the process evaluation, and the interprofessional collaboration in a clinical PHC context. The data from the process evaluation will increase and ease the possibility of implementation of a prevention program for NCDs in PHC. The risk of contamination between control and intervention is deemed minimal, as participants are recruited via social media in a large city.

ETHICS AND DISSEMINATION

For peer review only - http://bmjopen.bmj.com/site/about/guidelines.xhtml

An approval from the Swedish Ethical Review Authority, Sweden has been granted (Ref. numbers. 2015/834-31, 2016/2203-32, 2019/01444 and 2021-05902-02). Data management will be complying with the general data protection regulation, GDPR, and all data will be stored securely to protect the confidentiality. Participation in the study is not expected to lead to health risks or complications, and potential health consequences will be monitored. Participant who experiences any health-related problems during the study will be guided to contact their GP. Participants may choose to interrupt their participation in the study at any time. Researchers can also discontinue a participant's participation based on health issues, or reasons that might jeopardise that person's safety. Reasons for interruption will be recorded. For a summary of the consent form, see online supplemental 1.

The findings of the study will be published in peer-reviewed journals, and the results will be disseminated to participants, the public, PHC staff, and decision-makers through national and international conferences, as well as study-specific web pages.

AUTHOR CONTRIBUTIONS

AHP, EA, and SG conceived the original idea and outline of the study. AHP is implementing the protocol in primary healthcare settings, with regular dialog and review by CO, EÅ, GN, EA, and SG. CO, MH, GN, and EÅ contributed to the design of the study. AHP wrote the study protocol, together with EA and EJ. All authors discussed and commented on draft versions and approved the final version.

FUNDING STATEMENT

This work was funded by grants from FORTE, grant number 2020-00175. This funding source had no role in the design of this study and will not have any role during its execution, analyses, interpretation of the data, or decision to submit results.

COMPETING INTERESTS

The authors declare that they have no competing interests.

For peer review only

REFERENCES

1. Government S. Good quality, local health care—A primary care reform). http://www.sou.gov.se/wp-content/uploads/2018/06/Summary-SOU-2018.39_Final2.pdf; 2018. Report No.: SOU 2018:39.

2. Assembly. UNG. Transforming our world: The 2030 Agenda for sustainable development. 2016 2022-11-30. Available from: https://documents-dds-ny.un.org/doc/UNDOC/GEN/N15/291/89/PDF/N1529189.pdf?OpenElement

3. Habner J. Preventing stroke: Uneven progress. A global policy research programme. 2017.

4. Goldstein LB, Bushnell CD, Adams RJ, Appel LJ, Braun LT, Chaturvedi S, et al. Guidelines for the primary prevention of stroke: a guideline for healthcare professionals from the American Heart Association/American Stroke Association. Stroke. 2011;42(2):517–84.

5. Béjot Y, Bailly H, Durier J, Giroud M. Epidemiology of stroke in Europe and trends for the 21st century. La Presse Médicale. 2016;45(12, Part 2):e391–e8.

6. Welfare TNBoHa. Statistics on Stroke 2020. 2021. Contract No.: Art no. 2021-12-7644.

7. Organization) WWH. The top 10 causes of death 2020 [Available from: https://www.who.int/news-room/fact-sheets/detail/the-top-10-causes-of-death.

8. Chiuve SE, Rexrode KM, Spiegelman D, Logroscino G, Manson JE, Rimm EB. Primary prevention of stroke by healthy lifestyle. Circulation. 2008;118(9):947–54.

9. Eakin EG, Glasgow RE, Riley KM. Review of primary care-based physical activity intervention studies: effectiveness and implications for practice and future research. Database of Abstracts of Reviews of Effects (DARE): Quality-assessed Reviews [Internet]: Centre for Reviews and Dissemination (UK); 2000.

10. Weiss EC, Galuska DA, Khan LK, Gillespie C, Serdula MK. Weight regain in US adults who experienced substantial weight loss, 1999–2002. American journal of preventive medicine. 2007;33(1):34–40.

11. Hillsdon M, Thorogood M, White I, Foster C. Advising people to take more exercise is ineffective: a randomized controlled trial of physical activity promotion in primary care. Int J Epidemiol. 2002;31(4):808–15.

12. Sisti L, Dajko M, Campanella P, Shkurti E, Ricciardi W, De Waure C. The effect of multifactorial lifestyle interventions on cardiovascular risk factors: a systematic review and meta-analysis of trials conducted in the general population and high risk groups. Preventive medicine. 2018;109:82–97.

13. Lönnberg L, Ekblom-Bak E, Damberg M. Improved unhealthy lifestyle habits in patients with high cardiovascular risk: results from a structured lifestyle programme in primary care. Upsala journal of medical sciences. 2019;124(2):94–104.

14. Adibi S. Mobile Health: A Technology Road Map. Adibi S, editor: Springer; 2015.

15. Zonneveld M, Patomella A-H, Asaba E, Guidetti S. The use of information and communication technology in healthcare to improve participation in everyday life: a scoping review. Disability and Rehabilitation. 2019:1–8.

16. Chow CK, Ariyarathna N, Islam SM, Thiagalingam A, Redfern J. mHealth in Cardiovascular Health Care. Heart Lung Circ. 2016;25(8):802–7.

17. Owolabi MO, Thrift AG, Mahal A, Ishida M, Martins S, Johnson WD, et al. Primary stroke prevention worldwide: translating evidence into action. The Lancet Public Health. 2021.

18. Skivington K, Matthews L, Simpson SA, Craig P, Baird J, Blazeby JM, et al. Framework for the development and evaluation of complex interventions: gap analysis, workshop and consultation-informed update. 2021.

For peer review only - http://bmjopen.bmj.com/site/about/guidelines.xhtml

19. Jonsson H. A new direction in the conceptualization and categorization of occupation. Journal of occupational science. 2008;15(1):3–8.
20. Jonsson H, Josephsson S, Kielhofner G. Narratives and experience in an occupational transition: a longitudinal study of the retirement process. Am J Occup Ther. 2001;55(4):424–32.
21. Mälstam E, Asaba E, Åkesson E, Guidetti S, Patomella A-H. 'Weaving lifestyle habits': Complex pathways to health for persons at risk for stroke. Scandinavian journal of occupational therapy. 2021:1–13.
22. Asaba E, Bergström A, Patomella AH, Guidetti S. Engaging occupations among persons at risk for stroke: A health paradox. Scandinavian journal of occupational therapy. 2020:1–10.
23. Clark F, Azen SP, Zemke R, Jackson J, Carlson M, Mandel D, et al. Occupational therapy for independent-living older adults. A randomized controlled trial. JAMA. 1997;278(16):1321–6.
24. Clark F, Jackson J, Carlson M, Chou CP, Cherry BJ, Jordan-Marsh M, et al. Effectiveness of a lifestyle intervention in promoting the well-being of independently living older people: results of the Well Elderly 2 Randomised Controlled Trial. J Epidemiol Community Health. 2012;66(9):782–90.
25. Mountain G, Windle G, Hind D, Walters S, Keertharuth A, Chatters R, et al. A preventative lifestyle intervention for older adults (lifestyle matters): a randomised controlled trial. Age Ageing. 2017;46(4):627–34.
26. Moore GF, Audrey S, Barker M, Bond L, Bonell C, Hardeman W, et al. Process evaluation of complex interventions: Medical Research Council guidance. BMJ. 2015;350.
27. Patomella A-H, Farias L, Eriksson C, Guidetti S, Asaba E. Engagement in Everyday Activities for Prevention of Stroke: Feasibility of an mHealth-Supported Program for People with TIA. Healthcare. 2021;9(8):968.
28. Association NS. Stroke Risk Score Card 2018 [Available from: https://www.stroke.org/stroke-risk-scorecard-2018/.
29. Cup EH, op Reimer WS, Thijssen MC, van Kuyk-Minis M. Reliability and validity of the Canadian Occupational Performance Measure in stroke patients. Clinical rehabilitation. 2003;17(4):402.
30. Feigin V, Norrving B, Krishnamurthi R, Barker-Collo S, Wang W, Fu HUA, et al. Stroke Riskometer app: A new promising approach for primary stroke prevention and epidemiological research on stroke and other major non-communicable disorders (NCD) across the globe. International Journal of Stroke. 2015;10:101–2.
31. Medvedev O, Truong QC, Merkin A, Borotkanics R, Krishnamurthi R, Feigin V. Cross-cultural validation of the stroke riskometer using generalizability theory. Scientific Reports. 2021;11(1):1–10.
32. Law M, Baptiste S, McColl M, Opzoomer A, Polatajko H, Pollock N. The Canadian occupational performance measure: an outcome measure for occupational therapy. Can J Occup Ther. 1990;57(2):82–7.
33. Fugl-Meyer AR, Melin R, Fugl-Meyer KS. Life satisfaction in 18-to 64-year-old Swedes: in relation to gender, age, partner and immigrant status. Journal of rehabilitation medicine. 2002;34(5):239–46.
34. Burstrom K, Johannesson M, Diderichsen F. Swedish population health-related quality of life results using the EQ-5D. Qual Life Res. 2001;10(7):621-35.
35. Socialstyrelsen. Nationella riktlinjer för prevention och behandling vid ohälsosamma levnadsvanor. 2018.
36. Atler KE. User-perceived utility of the Daily Experiences of Pleasure, Productivity, and Restoration Profile. Can J Occup Ther. 2015;82(4):235–44.
37. Atler KE, Barney L, Moravec A, Sample PL, Fruhauf CA. The Daily Experiences of Pleasure, Productivity, and Restoration Profile: A case study: Etude de cas sur l'utilite du Daily Experiences of Pleasure, Productivity, and Restoration Profile. Can J Occup Ther. 2017;84(4-5):262–72.
38. Dearborn JL, McCullough LD. Perception of risk and knowledge of risk factors in women at high risk for stroke. Stroke. 2009;40(4):1181–6.

For peer review only - http://bmjopen.bmj.com/site/about/guidelines.xhtml

39. Prochaska JO, Redding CA, Evers KE. The transtheoretical model and stages of change. Health behavior: Theory, research, and practice. 2015:125–48.
40. Aguilar-Farías N, Brown WJ, Peeters GG. ActiGraph GT3X+ cut-points for identifying sedentary behaviour in older adults in free-living environments. Journal of science and medicine in sport. 2014;17(3):293–9.
41. Boutron I, Moher D, Altman DG, Schulz KF, Ravaud P. Extending the CONSORT statement to randomized trials of nonpharmacologic treatment: explanation and elaboration. Annals of internal medicine. 2008;148(4):295–309.
42. Chan A-W, Tetzlaff JM, Altman DG, Laupacis A, Gøtzsche PC, Krleža-Jerić K, et al. SPIRIT 2013 Statement: Defining standard protocol items for clinical trials. Ann Intern Med. 2013;158(3):200-207
43. Asaba E, Bergström A, Patomella A-H, Guidetti S. Engaging occupations among persons at risk for stroke: a health paradox. Scandinavian journal of occupational therapy. 2020:1-10.
44. Dahlgren C, Dackehag M, Wändell P, Rehnberg C. Determinants for use of direct-to-consumer telemedicine consultations in primary healthcare—a registry based total population study from Stockholm, Sweden. BMC family practice. 2021;22(1):1–10.
45. Mouton A, Cloes M. Efficacy of a web-based, center-based or combined physical activity intervention among older adults. Health Education Research. 2015;30(3):422–35.

Figure 1: An example of a checklist that shows the domains that the participants need to register in the app. Published with permission from ScientificMed Tech/Cuviva AB.

For peer review only

For peer review only - http://bmjopen.bmj.com/site/about/guidelines.xhtml

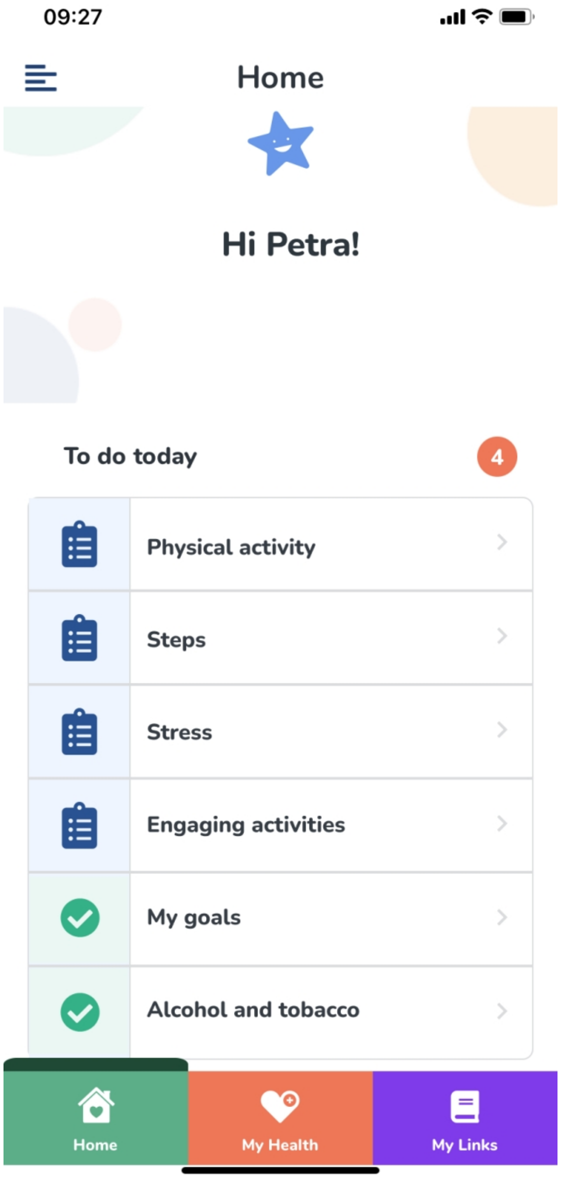

Figure 1: An example of a checklist that shows the domains that the participants need to register in the app. Published with permission from ScientificMed Tech/Cuviva AB.

751x1625mm (28 x 28 DPI)

Summarized constent form: Make My Day- Primary prevention of stroke using engaging everyday activities as a mediator of sustainable health

INFORMATION TO PARTICIPANTS IN THE PROJECT

We are asking if you are interested in participating in a research project. In this document you will receive information about the project and what participation entails.

WHAT IS THE PROJECT ABOUT AND WHY DO YOU WANT ME TO PARTICIPATE?

You are asked to participate in the study after responded to the advertisement for the study. To participate in the project, you must have at least three modifiable risk factors for stroke (examples of risk factors are high blood pressure, stroke in the family, low level of physical activity, overweight, smoking, high alcohol intake and unhealthy eating habits) and be 55-75 years old.

You also need to be able to participate in a health promotion program in a primary healthcare centre in the region. The program consists of physical meetings in a group at a nearby healthcare center (6 meetings over 10 weeks) and registration of lifestyle habits in a mobile phone app.

The study aims to evaluate a stroke prevention program that addresses modifiable risk factors for stroke. The research principal for the project is KI meaning that KI is the organization responsible for the study. The study has been approved by the Swedish Ethical Review Authority.

WHAT WILL HAPPEN DURING THE PROJECT?

You will initially be contacted by a researcher. You will then receive additional information about the study and be able to ask questions about what it means to participate.

If you want to participate in the study, you will meet with a researcher on three occasions to answer questions (at the start of the study, after eleven weeks and twelve months after the start of the study). You will also be able to answer questions via surveys digitally at home at your own pace. The questions will be about different aspects of your daily life with a focus on health. Each event on site will take about 1 hour and can take place either via a physical meeting or online. You will be offered to wear an activity tracker to measure your activity level. The activity tracker will record your physical movements (e.g., how long you spend in a sitting or standing position).

After the first meeting, you will be randomized to be a control for the study or to be in the prevention group. If you are randomized to the prevention group, you will be offered to participate in the 10-week prevention program at a healthcare center. We cannot control the randomization. You will be contacted after the randomization has been completed to find out which group you belong to.

If you are part of the prevention program, you will be asked to answer questions and to tell us how participating in the prevention program has worked for you. You will also be asked to identify a close relative who could consider answering a survey and being interviewed. You will not be excluded from participation in the study if you do not have a relative, it is not a requirement to be able to participate.

POSSIBLE CONSEQUENCES AND RISKS OF PARTICIPATING IN THE PROJECT

Participating in the project should not pose any risk to you. If you should experience any kind of discomfort during or after the completion of the questionnaires or measurements, you are asked to

For peer review only

discontinue your participation. If you experience any discomfort or ill health during the study, you are asked to contact your healthcare center and then contact the responsible researcher. The healthcare center to which you belong will offer customary support to you if needed throughout the course of the study (i.e., the customary support via your healthcare center is not replaced by study participation).

WHAT HAPPENS TO MY DATA?

A researcher will collect and record information about you. Your answers and your results will be processed so that unauthorized persons cannot access them. No personal information that can be linked to you will be used, analyzed, or provided to a third party. The information is protected by regulations on confidentiality, which means that no unauthorized person may access the information. According to the EU's data protection regulation (GDPR), you have the right to access, free of charge, the information about you that is handled in the study and where we got it from, and if necessary to have any errors corrected. If you are dissatisfied with the way your personal data is processed, you have the right to lodge a complaint with the Swedish Authority for Privacy Protection.

HOW DO I GET INFORMATION ABOUT THE RESULT OF THE PROJECT?

During data collection sessions, you will be informed about your own test results. The study results will be presented at group level in reports, conferences and in scientific publications.

INSURANCE AND COMPENSATION

When participating in medical research you are covered by patient insurance, which is based on the Patient Injury Act (SFS 1996:799). No compensation is paid for participation in the study, for example no compensation is paid for lost income.

PARTICIPATION IS VOLUNTARY

Your participation is voluntary, and you can choose to cancel your participation at any time. If you choose not to participate or wish to cancel your participation, you do not need to state why, and it will not affect your future care or treatment.

RESPONSIBLE FOR THE PROJECT

Those responsible for the project are xxx:

For peer review only - http://bmjopen.bmj.com/site/about/guidelines.xhtml

Consent to participate in the study

I have received oral and/or written information about the study and have had the opportunity to ask questions. I get to keep the written information.

• I agree to participate in the project: Stroke prevention – Development and evaluation of a person-centered, ICT-based intervention that supports a healthy activity pattern in everyday life in people who have an increased risk of suffering from a stroke

Location and Date	Signature
	Print name

For peer review only

Reporting checklist for protocol of a clinical trial.

Based on the SPIRIT guidelines.

Instructions to authors

Complete this checklist by entering the page numbers from your manuscript where readers will find each of the items listed below.

Your article may not currently address all the items on the checklist. Please modify your text to include the missing information. If you are certain that an item does not apply, please write "n/a" and provide a short explanation.

Upload your completed checklist as an extra file when you submit to a journal.

In your methods section, say that you used the SPIRITreporting guidelines, and cite them as:

Chan A-W, Tetzlaff JM, Altman DG, Laupacis A, Gøtzsche PC, Krleža-Jerić K, Hróbjartsson A, Mann H, Dickersin K, Berlin J, Doré C, Parulekar W, Summerskill W, Groves T, Schulz K, Sox H, Rockhold FW, Rennie D, Moher D. SPIRIT 2013 Statement: Defining standard protocol items for clinical trials. Ann Intern Med. 2013;158(3):200-207

		Reporting Item	Page Number
**Administrative information**			
Title	#1	Descriptive title identifying the study design, population, interventions, and, if applicable, trial acronym	1
Trial registration	#2a	Trial identifier and registry name. If not yet registered, name of intended registry	2
Trial registration: data set	#2b	All items from the World Health Organization Trial Registration Data Set	N/A a registration has not been done
Protocol version	#3	Date and version identifier	2
Funding	#4	Sources and types of financial, material, and other support	20

For peer review only

Roles and responsibilities: contributorship	#5a	Names, affiliations, and roles of protocol contributors	20
Roles and responsibilities: sponsor contact information	#5b	Name and contact information for the trial sponsor	N/A, no trial sponsor
Roles and responsibilities: sponsor and funder	#5c	Role of study sponsor and funders, if any, in study design; collection, management, analysis, and interpretation of data; writing of the report; and the decision to submit the report for publication, including whether they will have ultimate authority over any of these activities	N/A
Roles and responsibilities: committees	#5d	Composition, roles, and responsibilities of the coordinating centre, steering committee, endpoint adjudication committee, data management team, and other individuals or groups overseeing the trial, if applicable (see Item 21a for data monitoring committee)	N/A
**Introduction**			
Background and rationale	#6a	Description of research question and justification for undertaking the trial, including summary of relevant studies (published and unpublished) examining benefits and harms for each intervention	7-9
Background and rationale: choice of comparators	#6b	Explanation for choice of comparators	8-9
Objectives	#7	Specific objectives or hypotheses	9
Trial design	#8	Description of trial design including type of trial (eg, parallel group, crossover, factorial, single group), allocation ratio, and framework (eg, superiority, equivalence, non-inferiority, exploratory)	9
**Methods: Participants,**			

For peer review only

**interventions, and outcomes**			
Study setting	#9	Description of study settings (eg, community clinic, academic hospital) and list of countries where data will be collected. Reference to where list of study sites can be obtained	9
Eligibility criteria	#10	Inclusion and exclusion criteria for participants. If applicable, eligibility criteria for study centres and individuals who will perform the interventions (eg, surgeons, psychotherapists)	10-11
Interventions: description	#11a	Interventions for each group with sufficient detail to allow replication, including how and when they will be administered	11-12
Interventions: modifications	#11b	Criteria for discontinuing or modifying allocated interventions for a given trial participant (eg, drug dose change in response to harms, participant request, or improving / worsening disease)	N/A
Interventions: adherance	#11c	Strategies to improve adherence to intervention protocols, and any procedures for monitoring adherence (eg, drug tablet return; laboratory tests)	N/A
Interventions: concomitant care	#11d	Relevant concomitant care and interventions that are permitted or prohibited during the trial	N/A
Outcomes	#12	Primary, secondary, and other outcomes, including the specific measurement variable (eg, systolic blood pressure), analysis metric (eg, change from baseline, final value, time to event), method of aggregation (eg, median, proportion), and time point for each outcome. Explanation of the clinical relevance of chosen efficacy and harm outcomes is strongly recommended	13-14
Participant timeline	#13	Time schedule of enrolment, interventions (including any run-ins and washouts), assessments, and visits for participants. A schematic diagram is highly recommended (see Figure)	15

For peer review only

Sample size	#14	Estimated number of participants needed to achieve study objectives and how it was determined, including clinical and statistical assumptions supporting any sample size calculations	10
Recruitment	#15	Strategies for achieving adequate participant enrolment to reach target sample size	10
**Methods: Assignment of interventions (for controlled trials)**			
Allocation: sequence generation	#16a	Method of generating the allocation sequence (eg, computer-generated random numbers), and list of any factors for stratification. To reduce predictability of a random sequence, details of any planned restriction (eg, blocking) should be provided in a separate document that is unavailable to those who enrol participants or assign interventions	10-11
Allocation concealment mechanism	#16b	Mechanism of implementing the allocation sequence (eg, central telephone; sequentially numbered, opaque, sealed envelopes), describing any steps to conceal the sequence until interventions are assigned	11
Allocation: implementation	#16c	Who will generate the allocation sequence, who will enrol participants, and who will assign participants to interventions	N/A not been decided
Blinding (masking)	#17a	Who will be blinded after assignment to interventions (eg, trial participants, care providers, outcome assessors, data analysts), and how	10
Blinding (masking): emergency unblinding	#17b	If blinded, circumstances under which unblinding is permissible, and procedure for revealing a participant's allocated intervention during the trial	N/A
**Methods: Data collection,**			

For peer review only

**management, and analysis**			
Data collection plan	#18a	Plans for assessment and collection of outcome, baseline, and other trial data, including any related processes to promote data quality (eg, duplicate 6measurements, training of assessors) and a description of study instruments (eg, questionnaires, laboratory tests) along with their reliability and validity, if known. Reference to where data collection forms can be found, if not in the protocol	13-15
Data collection plan: retention	#18b	Plans to promote participant retention and complete follow-up, including list of any outcome data to be collected for participants who discontinue or deviate from intervention protocols	N/A not been decided
Data management	#19	Plans for data entry, coding, security, and storage, including any related processes to promote data quality (eg, double data entry; range checks for data values). Reference to where details of data management procedures can be found, if not in the protocol	15
Statistics: outcomes	#20a	Statistical methods for analysing primary and secondary outcomes. Reference to where other details of the statistical analysis plan can be found, if not in the protocol	17-18
Statistics: additional analyses	#20b	Methods for any additional analyses (eg, subgroup and adjusted analyses)	N/A
Statistics: analysis population and missing data	#20c	Definition of analysis population relating to protocol non-adherence (eg, as randomised analysis), and any statistical methods to handle missing data (eg, multiple imputation)	Not decided
**Methods: Monitoring**			
Data monitoring: formal committee	#21a	Composition of data monitoring committee (DMC); summary of its role and reporting structure;	N/A

For peer review only

		statement of whether it is independent from the sponsor and competing interests; and reference to where further details about its charter can be found, if not in the protocol. Alternatively, an explanation of why a DMC is not needed	
Data monitoring: interim analysis	#21b	Description of any interim analyses and stopping guidelines, including who will have access to these interim results and make the final decision to terminate the trial	N/A
Harms	#22	Plans for collecting, assessing, reporting, and managing solicited and spontaneously reported adverse events and other unintended effects of trial interventions or trial conduct	N/A
Auditing	#23	Frequency and procedures for auditing trial conduct, if any, and whether the process will be independent from investigators and the sponsor	N/A
**Ethics and dissemination**			
Research ethics approval	#24	Plans for seeking research ethics committee / institutional review board (REC / IRB) approval	19
Protocol amendments	#25	Plans for communicating important protocol modifications (eg, changes to eligibility criteria, outcomes, analyses) to relevant parties (eg, investigators, REC / IRBs, trial participants, trial registries, journals, regulators)	N/A
Consent or assent	#26a	Who will obtain informed consent or assent from potential trial participants or authorised surrogates, and how (see Item 32)	11
Consent or assent: ancillary studies	#26b	Additional consent provisions for collection and use of participant data and biological specimens in ancillary studies, if applicable	N/A
Confidentiality	#27	How personal information about potential and enrolled participants will be collected, shared, and maintained in order to protect confidentiality before, during, and after the trial	19

For peer review only

Declaration of interests	#28	Financial and other competing interests for principal investigators for the overall trial and each study site	20
Data access	#29	Statement of who will have access to the final trial dataset, and disclosure of contractual agreements that limit such access for investigators	19, has been informed to the ethics review board
Ancillary and post trial care	#30	Provisions, if any, for ancillary and post-trial care, and for compensation to those who suffer harm from trial participation	None
Dissemination policy: trial results	#31a	Plans for investigators and sponsor to communicate trial results to participants, healthcare professionals, the public, and other relevant groups (eg, via publication, reporting in results databases, or other data sharing arrangements), including any publication restrictions	20
Dissemination policy: authorship	#31b	Authorship eligibility guidelines and any intended use of professional writers	20
Dissemination policy: reproducible research	#31c	Plans, if any, for granting public access to the full protocol, participant-level dataset, and statistical code	None
**Appendices**			
Informed consent materials	#32	Model consent form and other related documentation given to participants and authorised surrogates	An example is provided
Biological specimens	#33	Plans for collection, laboratory evaluation, and storage of biological specimens for genetic or molecular analysis in the current trial and for future use in ancillary studies, if applicable	N/A

None The SPIRIT checklist is distributed under the terms of the Creative Commons Attribution License CC-BY-ND 3.0. This checklist can be completed online using https://www.goodreports.org/, a tool made by the EQUATOR Network in collaboration with Penelope.ai

For peer review only
